# *Legionella* effector protein SidG disrupts host cytoskeleton via targeting Arp2/3 complex

**DOI:** 10.1371/journal.ppat.1013957

**Published:** 2026-02-09

**Authors:** Jiayang Liu, Siyao Liu, Rundong Shu, Kelong Ma, Qian Lu, Jinli Ge, Hongtao Liu, Jiaqi Fu, Jiazhang Qiu

**Affiliations:** 1 State Key Laboratory for Diagnosis and Treatment of Severe Zoonotic Infectious Diseases, Key Laboratory for Zoonosis Research of the Ministry of Education, College of Veterinary Medicine, Jilin University, Changchun, China; 2 Department of Respiratory Medicine, Center of Pathogen Biology and Infectious Diseases, Key Laboratory of Organ Regeneration and Transplantation of The Ministry of Education, State Key Laboratory for Diagnosis and Treatment of Severe Zoonotic Infectious Diseases, The First Hospital of Jilin University, Changchun, China; Gifu University, JAPAN

## Abstract

*Legionella pneumophila* is a facultative intracellular bacterial pathogen capable of surviving and replicating within host cells, including macrophages and protozoans. It employs the Dot/Icm type IV secretion system (T4SS) to inject over 330 effector proteins into host cells, manipulating various cellular processes to facilitate infection. Characterizing the functions of these effectors is crucial to deciphering the pathogenesis of *L. pneumophila*. In this study, we identified SidG as an effector containing a Cys-His-Asp triad, whose functional state is strictly gated by its interaction with the cellular GTPase Rac1, particularly via its C-terminal domain. Rac1-activated SidG then utilizes an acidic (A) domain to target the Arp2/3 complex, the key regulator of actin nucleation. Importantly, SidG disrupts cytoskeletal architecture via both Rac1- and Arp2/3-dependent mechanisms. During *L. pneumophila* infection, SidG is crucial to promote efficient bacterial invasion of host cells in a Cys-His-Asp motif-dependent manner. Together, our study elucidates a sophisticated pathogenic mechanism where a bacterial effector co-opts a host GTPase to allosterically regulate its function towards the Arp2/3 complex, thereby facilitating bacterial entry into host cells.

## Author summary

To invade and proliferate within human immune cells, *Legionella* employs a sophisticated infection strategy to inject over 330 bacterial effector proteins directly into host cells to manipulate cellular processes. Among these effectors, we found that SidG plays a critical role in disrupting the host cytoskeleton, a network of protein filaments essential for maintaining cell structure and motility. Our results revealed that SidG functions as a tightly regulated molecular machine that remains dormant until triggered. Specifically, SidG initially binds to the host GTPase Rac1, which acts as a molecular switch to release SidG from its inactive state. Upon activation, SidG then targets the Arp2/3 complex and disrupts actin network. This dual regulatory mechanism allows *Legionella* to manipulate host cell architecture and facilitate bacterial entry into cells. Our findings uncover a sophisticated strategy in which pathogens hijack host cellular machinery to establish infection, providing new insights into *Legionella* pathogenesis and potential therapeutic targets.

## Introduction

*Legionella pneumophila* is a Gram-negative facultative intracellular bacterial pathogen that causes Legionnaires’ disease, a severe and potentially fatal pneumonia, as well as Pontiac fever, a self-limited febrile illness [1]. *L. pneumophila* is primarily found in freshwater and man-made water systems (e.g., lakes, rivers, streams, and cooling towers), where it parasitizes free-living protozoa including distinct species of amoebae [2]. Transmission of *L. pneumophila* typically occurs through inhalation of contaminated water aerosols by immunocompromised or otherwise susceptible hosts [3]*.* In the human lung, *L. pneumophila* invades and replicates within alveolar macrophages, and to a lesser extent in epithelial cells, utilizing evolutionarily conserved mechanisms similar to those employed during its environmental colonization of amoebae [4]. *Legionella* infection in humans has historically been considered a dead-end event with no evidence of human-to-human transmission. However, one probable case of human-to-human transmission has recently been reported, which might represent a new mechanism of *L. pneumophila* transmission [5].

The intracellular life cycle of *L. pneumophila* has been well-characterized after decades of investigation. Following internalization by phagocytic cells via conventional or coiling phagocytosis, *L. pneumophila* subverts lysosomal degradation and establishes a specialized replicative niche-the *Legionella*-containing vacuole (LCV) within the host cells [6]. Unlike compartments harboring non-pathogenic bacteria, the LCV maintains neutral pH and acquires a unique membrane identity through selective recruitment of cytoplasmic vesicles enriched with specific lipids and proteins [7]. The biogenesis and maturation of LCV strictly relies on the Dot/Icm type IV secretion system (T4SS) [8]. The multi-protein machinery is composed of 27 proteins and responsible for the translocation of more than 330 effector proteins from the bacteria directly into the host cells [4]. Upon translocation into the host cytoplasm, these bacterial effectors target various host substrates, altering signaling pathways to manipulate multiple host responses, which is essential for *L. pneumophila* to establish successful infection [8]. Therefore, identifying the biochemical functions as well as the host targets of individual effectors is crucial for deciphering the molecular mechanisms of pathogenesis. However, despite intensive investigation, biochemical characterization has been achieved for only a small fraction of the roughly 330 Dot/Icm effector proteins.

The actin cytoskeleton is a dynamic and tightly regulated network essential for maintaining cell structure, motility, and membrane trafficking [9]. Due to its central role in cellular function, it is not surprising that many bacterial pathogens have evolved sophisticated strategies to manipulate host actin dynamics, facilitating invasion, intracellular movement, immune evasion, and replication [10]. For instance, *Salmonella* T3SS effector proteins SopE and SopE2 function as guanine nucleotide exchange factors to activate CDC42 and Rac small GTPase, triggering downstream signaling cascades that remodel the actin cytoskeleton, promoting *Salmonella* invasion into host cells [11]. The C2 toxin from *Clostridium botulinum* acts as an ADP-ribosyltransferase to modify Arg-177 of actin, thus blocking its polymerization [12].

SidG (Lpg1355) is a *Legionella* Dot/Icm substrate that utilizes an IcmSW-dependent mechanism for translocation [13]. Little is known about its biochemical function as well as biological roles in *Legionella* pathogenesis. In this study, we report that SidG hijacks host actin dynamics by modulating the Arp2/3 complex, thereby inducing cytoskeletal network remodeling. Mechanistically, we demonstrate that SidG activity is controlled by a molecular switch. Its C-terminal domain binds the host GTPase Rac1, which functions as an allosteric activator. Such interaction allows the Cys-His-Asp triad to target the Arp2/3 complex, with the ArpC3 subunit playing an essential role in this process. Our findings expand the repertoire of pathogen-encoded effector proteins critical for hijacking the host actin cytoskeletal network.

## Results

### The Cys-His-Asp motif is critical for the yeast toxicity of SidG

**Fig 1:**
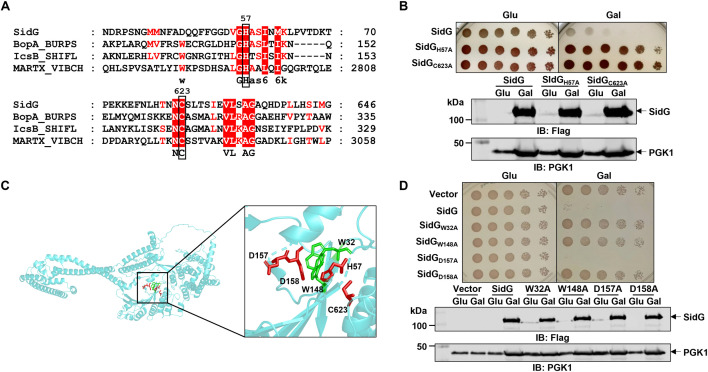
Effector protein SidG harbors a Cys-His-Asp triad which is critical for its yeast toxicity. **A** Sequence alignment of SidG (Lpg1355) with BopA from *Burkholderia pseudomallei*, IcsB from *Shigella flexneri*, and MARTX toxins from *Vibrio cholerae*. Alignment was generated by Clustal Omega and GeneDoc. Identical residues are shaded in red, and similar residues are shown in red font. **B** Yeast toxicity of wild-type SidG, SidG_H57A_, and SidG_C623A_. The W303 strain expressing the indicated Flag-SidG constructs from a galactose-inducible promoter was serially diluted and spotted onto glucose (promoter repressed) or galactose (promoter induced) plates. Expression of SidG in these strains was probed by immunoblot using anti-Flag antibodies. PGK1 (3-phosphoglyceric phosphokinase) served as a loading control. **C** AlphaFold-predicted structure of SidG. The Cys-His-Asp triad and the key tryptophan residues are highlighted. **D** Yeast toxicity of SidG constructs with mutations in W32A, W148A, D157A, or D158A. The experiment was performed as described in **(B)**. Data shown in **B** and **D** are representative from three independent experiments.

SidG is a 974 amino-acid Dot/Icm effector protein exhibiting no significant primary sequence homology to proteins of known function. Furthermore, bioinformatics analysis based on remote secondary structure homology using pairwise comparison of profile hidden Markov models (HHpred) failed to identify any confident matches. We thus utilized HMMER to analyze amino acid sequence conservation of SidG, which allowed us to identify W32, G56, H57, W148, N622, and C623 of SidG as high conservation residues (S1 Fig). The high sequence conservation observed at residue pairs G56-H57 and N622-C623 suggests their potential involvement in a conserved structural or functional motif, such as the Cys-His-Asp motif. Indeed, this hypothesis was further supported by alignment of SidG with Cys-His-Asp motif-containing proteins including the *Burkholderia pseudomallei* T3SS effector BopA, *Shigella flexneri* effector IcsB, and *Vibrio* species multifunctional autoprocessing repeats-in-toxin (MARTX) toxins (Fig 1A) [14–16]. Consistent with a recent study [17], SidG is toxic to yeast, and this toxicity was completely abolished by individual mutation of H57 or C623 to alanine (Fig 1B). To identify the Asp residue constituting the Cys-His-Asp motif in SidG, we analyzed its AlphaFold-predicted structure (AF-Q5ZVT5-F1-v4). Notably, two Asp residues (D157 and D158) were positioned in spatial proximity to H57 and C623 (Fig 1C). Subsequent yeast toxicity assay demonstrated that mutation of D158, but not D157, abrogated SidG’s toxicity (Fig 1D). Collectively, these results confirmed that H57, D158, and C623 constitute the Cys-His-Asp motif of SidG. Unlike canonical Cys-His-Asp motifs where these residues are contiguous in the primary sequence, the SidG Cys-His-Asp motif is spatially assembled through protein folding to create a functional pocket. In addition, alanine substitution of W32 and W148, two residues positioned near H57 and D158, respectively, similarly eliminated SidG toxicity (Fig 1D), suggesting that these tryptophan residues are likely involved in maintaining the structure of the Cys-His-Asp pocket (Fig 1C).

Despite primary sequence variation, the Cys-His-Asp motif shows remarkable conservation among *Legionella* SidG orthologs (S2A and S2B Fig). Ectopic expression of two SidG orthologs, SidG_*LS*_ (from *Legionella sainthelensi*) and SidG_*LB*_ (from *Legionella brunensis*), proved lethal to yeast (S2C Fig). Similarly, alanine substitution of any individual Cys-His-Asp residue in SidG_*LS*_ abolished its toxic effect on yeast (S2D Fig). These results demonstrate that SidG and its orthologs maintain an evolutionarily conserved Cys-His-Asp triad whose activity likely depends on proper three-dimensional structural organization.

### The C-terminal domain of SidG mediates specific interaction with Rac1

**Fig 2:**
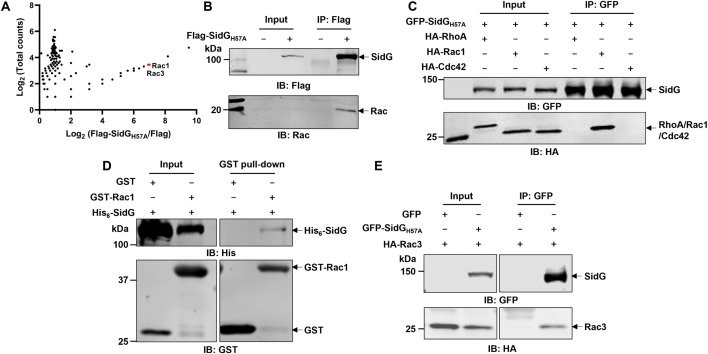
The Rac1 GTPase is identified as a specific binding partner for SidG. **A** Identification of SidG interacting proteins. Flag-SidG_H57A_ was expressed in HEK293T cell lysates and enriched by anti-Flag immunoprecipitation. Immunoprecipitated proteins were identified by mass spectrometric analysis. Rac1 and Rac3 are highlighted in red. **B** Validation of the interaction between Flag-SidG_H57A_ and endogenous Rac proteins by co-immunoprecipitation. Lysates of Flag-SidG_H57A_ expressing HEK293T cells were immunoprecipitated with anti-Flag beads followed by immunoblotting with anti-Flag or anti-Rac antibodies. **C** Assessment of the interaction between various Rho GTPases and SidG. HA-tagged RhoA, Rac1, and Cdc42 were co-expressed with GFP-SidG_H57A_ in HEK293T cells. After immunoprecipitation by anti-GFP beads, the beads-bound proteins were analysed by immunoblotting with anti-GFP and anti-HA antibodies. **D** SidG binds to Rac1 *in vitro* by GST pull-down assay. GST-Rac1-bound SidG was detected by immunoblotting with anti-His_6_ antibodies. **E** Co-immunoprecipitation of HA-Rac3 co-expressed with GFP-SidG_H57A_ in HEK293T cells, anti-GFP immunoprecipitation was further analysed by immunoblotting with anti-GFP and anti-HA antibodies. Data shown in **B**-**E** are representative from three independent experiments.

Next, we attempted to identify potential binding partners of SidG using immunoprecipitation (IP) followed by mass spectrometry analysis. Despite extensive efforts, we were unable to detect decent expression of wild-type SidG in distinct mammalian cell lines. Instead, we expressed and enriched Flag-SidG_H57A_ in HEK293T cells by anti-Flag IP. Flag-SidG_H57A_ bound proteins were analyzed by LC-MS/MS. Notably, Rac1 and Rac3 were identified at high score in this analysis (Figs 2A and S3A). The potential interaction between SidG_H57A_ and Rac small GTPase was validated by Western-blot analysis of the immunoprecipitates with Rac antibody recognizing both Rac1 and Rac3 (Fig 2B). Furthermore, co-immunoprecipitation experiments using cell lysates co-expressing GFP-SidG_H57A_ and HA-tagged Rac1/Rac3/RhoA/CDC42, followed by Western-blot analysis using anti-HA antibody, revealed that SidG specifically interacts with Rac1 and Rac3 but not with other Rho family small GTPases, including RhoA or CDC42 (Fig 2C and 2E). Co-expression of GST-Rac1 and His_6_-SidG in *E. coli* also revealed direct association between these two proteins (Fig 2D). Small GTPases cycles between a GTP-bound active and a GDP-bound inactive form catalyzed by GEF and GAP, respectively [18]. To determine whether SidG has binding preference for one of these two forms, we co-expressed GFP-SidG_H57A_ with either Rac1_Q61L_, a dominant-positive mutant or Rac1_T17N_, a dominant-negative mutant in HEK293T cell. A slight preference of SidG for the Rac1-GTP form was observed by Western-blot analysis (S3B and S3C Fig). However, none of Rac1 WT, Rac1_Q61L_ (GTP-bound form), or Rac1_T17N_ (GDP-bound form) could suppress the yeast growth defect caused by SidG (S4A Fig). Furthermore, incubation of GFP-SidG with HA-Rac1 did not affect Rac1 binding to the p21-binding domain (PBD) of p21-activated protein kinase (PAK) (S4B Fig), suggesting that SidG cannot disrupt the activation of Rac1.

We utilized AlphaFold to perform a structural prediction of the interaction, which revealed a distinct interaction interface between Rac1 and the C-terminus of SidG (S5A Fig). A series of SidG truncation mutants were constructed and co-expressed with HA-Rac1 in HEK293T cells. After anti-HA IP and Western-blot analysis, the C-terminal region of SidG_700-973_ was sufficient to interact with Rac1, with a binding strength comparable to that of full-length SidG (S5B Fig). These findings support a model in which Rac1 serves not as a direct target, but rather as an essential upstream activator of SidG, potentially through allosteric regulation of its activity.

### Host Rac1 is a prerequisite for the function of SidG

**Fig 3:**
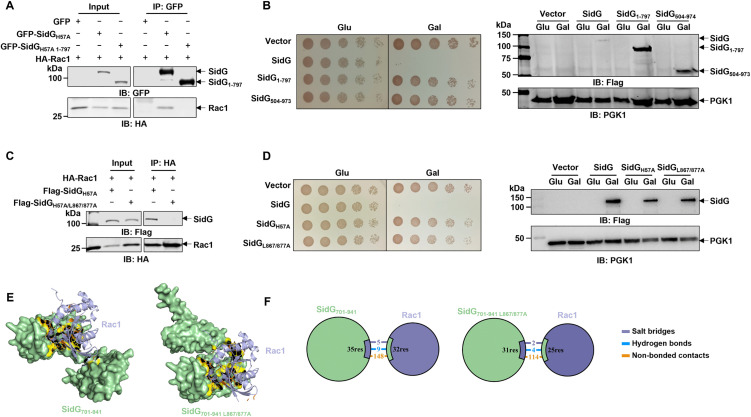
Disruption of the SidG-Rac1 interface abrogates SidG toxicity. **A**, **B** The C-terminal region of SidG is required for both Rac1 binding and yeast toxicity. Co-immunoprecipitation analysis of the interaction between HA-Rac1 and GFP-SidG_1-797_. Lysates from transfected HEK293T cells were subjected to immunoprecipitation with an anti-GFP antibody followed by immunoblotting with anti-GFP and anti-HA antibodies **(A)**. Yeast toxicity assay of wild-type SidG, SidG_1-797_, and SidG_504-973_ was performed as in Fig 1B. Right panel shows the immunoblots for protein expression levels **(B)**. **C**, **D** Specific residues in the C-terminus of SidG are critical for Rac1 binding and yeast toxicity. Co-immunoprecipitation analysis of the interaction between HA-Rac1 and Flag-tagged SidG_H57A_ or SidG_H57A/L867A/L877A_ in transfected HEK293T cells **(C)**. Yeast toxicity assay of the SidG_L867A/L877A_. Right panel shows an immunoblot for protein expression levels **(D)**. **E** AlphaFold-predicted structural models for SidG_797-941_ (left) or SidG_797-941 L867A/L877A_ (right) in complex with Rac1. **F** 2D diagrams were created by PDBsum to summarize the predicted molecular contacts between Rac1 and SidG_797-941_ (left) or SidG_797-941 L867A/L877A_ (right). Data shown in **A**-**D** are representative from three independent experiments.

To further determine the roles of SidG-Rac1 interaction, we first constructed a SidG_1-797_ truncation mutant which contains the intact Cys-His-Asp motif but lacks the C-terminal domain. Indeed, SidG_1-797_ failed to interact with the Rac1 protein (Fig 3A) and lost the ability to cause a yeast growth defect (Fig 3B). This finding suggests that Rac1 binding does not simply relieve autoinhibition but is strictly required for the functional activation of SidG. We then checked the yeast toxicity of the SidG_504-973_ truncation mutant that retains full Rac1 binding ability but lacks the intact Cys-His-Asp motif, which also showed a loss of the yeast growth defect phenotype (Fig 3B). Considering that the truncation mutagenesis may affect the structural integrity of SidG protein, we aimed to identify critical residues that mediate SidG-Rac1 interaction. We used LigPlot^+^ to analyze the interaction interface based on the SidG-Rac1 complex structure predicted by AlphaFold, which identified three key residues in SidG: E821, D836, and D883 (S6A Fig). The sequential introduction of single, double, and triple mutations at these residues induced stepwise attenuation of the SidG-Rac1 interaction (S6B Fig). These results not only confirm the critical involvement of E821, D836, and D883 in mediating the interaction, but also corroborate the structural fidelity of the computationally predicted SidG-Rac1 binding model. Although the SidG_H57A/E821A/D883A/D836A_ mutant showed the weakest binding affinity to Rac1, complete ablation of the SidG-Rac1 interaction remained unobtainable (S6B Fig). We further applied a hydroxylamine-based random mutagenesis strategy to screen for additional residues that affect the interaction and identified three additional residues: L867, L877, and V927. Individual substitution of each residue to alanine all displayed markable reduction in its binding to Rac1 (S6C Fig). Furthermore, simultaneous alanine substitution at residues L867 and L877 (SidG_L867A/L877A_) completely abrogated the SidG-Rac1 interaction (Fig 3C). Consistent with this molecular disruption, the double mutant failed to inhibit yeast growth (Fig 3D). Structural modeling predicts that the C-terminal domain of SidG adopts a pincer-like conformation that fully encapsulates Rac1, whereas the SidG_L867A/L877A_ double mutant maintains only a single binding interface (Fig 3E and 3F). Importantly, analysis of the predicted secondary structure topology reveals that this mutant retains overall structural integrity (S6D Fig), demonstrating that L867A/L877A mutations specifically impair interfacial contacts. To further establish that SidG activity is strictly dependent on Rac1-family GTPases, we investigated the role of Rho5, the yeast homolog of mammalian Rac1. Superposition of the AlphaFold models revealed strong conservation of the GTPase core domain between Rho5 and human Rac1 (S7A Fig). We therefore generated a homozygous *RHO5* deletion strain (*rho5*∆::*TRP1*), which was validated by sequencing and RT-qPCR (S7B and S7C Fig). Notably, the severe growth inhibition caused by SidG in wild-type yeast was abolished in the *RHO5* deletion strain (S7D Fig). Furthermore, ectopic expression of human Rac1 in this background fully restored SidG-mediated toxicity (S7D Fig). Collectively, our data establish the SidG-Rac1 interaction as an essential molecular switch governing effector activation, with its engagement serving as a strict prerequisite for SidG-mediated yeast toxicity.

### SidG hijacks the Arp2/3 complex by mimicking eukaryotic regulatory protein via an A domain

**Fig 4:**
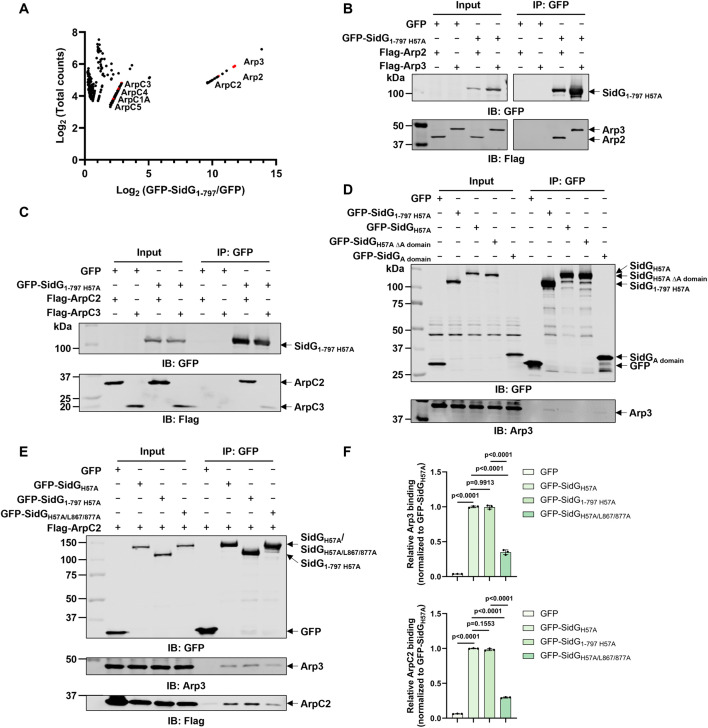
The A domain of SidG mediates interaction with the Arp2/3 complex. **A** Lysates from HEK293T cells expressing GFP-SidG_1-797_ were subjected to anti-GFP immunoprecipitation followed by mass spectrometric analysis. Subunits of the Arp2/3 complex are highlighted in red. **B**, **C** Validation of the interaction between GFP-SidG_1-797 H57A_ and Flag-tagged Arp2/Arp3 (**B**) or ArpC2/ArpC3 (**C**) by co-immunoprecipitation of transfected HEK293T cells with anti-GFP beads. Immunoblotting was performed with antibodies specific for GFP and Flag. **D** The A domain of SidG is critical for its association with Arp3. Lysates from HEK293T cells expressing GFP-SidG_H57A_, GFP-SidG_H57A ∆A domain_, or GFP-SidG_A domain_ were subjected to anti-GFP immunoprecipitation followed by immunoblotting with endogenous Arp3. **E, F** Rac1 binding is required to expose the Arp2/3-binding interface. **(E)** Co-immunoprecipitation analysis of the interaction between Arp3/Flag-ArpC2 and the indicated GFP-SidG variants in HEK293T cells. **(F)** Quantification of Arp3 and ArpC2 relative binding levels shown in **(E)**. Band intensities were normalized to the SidG_H57A_ control. Data are mean ± SD (n = 3) and analyzed by one-way ANOVA. Data shown in **B**-**E** are representative from three independent experiments.

To further identify the downstream targets of SidG, we performed another IP-mass spectrometry analysis using the N-terminal fragment GFP-SidG_1-797_ as bait. This strategy aimed to functionally isolate the target-binding domain from the C-terminal regulatory region. Among the proteins specifically enriched by GFP-SidG_1-797_, all seven Arp2/3 complex subunits were identified with high-confidence (Fig 4A). We then confirmed Arp2/3 complex as targets of SidG by transfecting mammalian cells to express GFP-SidG_1-797 H57A_ and Flag-Arp2 and examined their interactions by anti-GFP IP. Notably, Flag-Arp2 was detected in anti-GFP precipitates from cell lysates co-expressing GFP-SidG_1-797 H57A_ but not GFP (Fig 4B). In line with mass spectrometric results, we could also detect interactions between GFP-SidG_1-797 H57A_ and other Arp2/3 complex subunits (Arp3, ArpC2 and ArpC3) by co-immunoprecipitation (Fig 4B-D). To distinguish between subunit-specific and holocomplex recognition, we performed binding assays using *E. coli* lysates. While His-Flag-Rac1 specifically pulled down full-length GST-SidG, neither full-length SidG nor SidG_1-797_ bound to any individually expressed Arp2/3 subunit (S8 Fig). These results demonstrate that SidG specifically recognizes the assembled Arp2/3 holocomplex.

**Fig 5:**
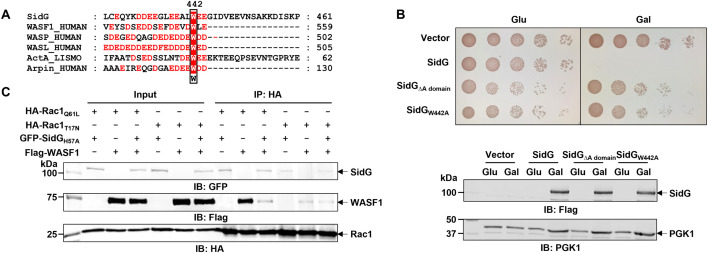
The A domain embedded in SidG is essential for its function. **A** Sequence alignment of SidG with the A domains of eukaryotic Arp2/3 regulators and *Listeria monocytogenes* ActA. Conserved acidic residues are shown in red and the critical tryptophan residue is boxed. **B** Yeast toxicity assays of SidG_∆A domain_ and SidG_W442A_ were performed as in Fig 1B. **C** Competitive co-immunoprecipitation assay for SidG and WASF1 binding to active Rac1 (Rac1_Q61L_) in transfected HEK293T cells. Immunoprecipitated proteins by anti-HA beads were further probed for antibodies specific for GFP, Flag, and HA. Data shown in **B** and **C** are representative from three independent experiments.

To elucidate the molecular basis of SidG-Arp2/3 complex interaction, we performed comprehensive sequence analysis of SidG and identified a conserved Asp/Glu-rich acidic region (residues 432–444) (Fig 5A). This acidic motif, designated as the A domain, represents a characteristic structural feature shared by both activating and inhibitory regulators of the Arp2/3 complex [19]. To investigate the potential role of the A domain in interaction of SidG with the Arp2/3 complex, we constructed a SidG_ΔA domain_ mutant with complete deletion of the acidic cluster. Subsequent experiments revealed that this mutant failed to bind Arp2/3 complex component Arp3 (Fig 4D). Moreover, the truncation mutant SidG_A domain_ containing only the A domain was sufficient to precipitate Arp3 (Fig 4D), establishing this acidic motif as both necessary and sufficient for the SidG-Arp2/3 complex engagement. However, access to this domain is strictly regulated by an auto-inhibitory mechanism by the C-terminal domain. Consistent with this, the Rac1-binding-deficient mutant (SidG_H57A/L867A/L877A_) exhibited significantly impaired interaction with the Arp2/3 complex. In contrast, the C-terminally truncated SidG_1-797 H57A_ retained strong binding affinity (Fig 4E and 4F), confirming that the C-terminus masks the A domain in the absence of Rac1. Crucially, the SidG_ΔA domain_ mutant completely lost the ability to inhibit yeast growth (Fig 5B). This establishes the A domain as the key functional module exposed by Rac1-mediated activation. The sequence alignment analysis revealed that the A domain of SidG also contains a conserved Trp residue (W442) (Fig 5A). Functional analysis of a W442A substitution mutant showed complete loss of yeast growth inhibition (Fig 5B), demonstrating that this residue is essential for SidG activity.

Based on these findings, we propose that Rac1 serves as an allosteric co-factor of SidG, enabling its subsequent action toward the Arp2/3 complex. This Rac1-dependent regulatory mechanism of SidG mirrors the activation paradigm observed in canonical nucleation-promoting factors (NPFs) [20]. To further investigate this functional similarity, we hypothesized that SidG might compete with WASF1 (WASP-family verprolin-homologous protein 1), a prototypical NPF [21], for Rac1 binding. Remarkably, co-expression of SidG significantly attenuated the interaction between WASF1 and the GTP-bound active form of Rac1 (Rac1_Q61L_), while having no effect on its binding to the GDP-bound inactive form (Fig 5C). These findings suggest that SidG could interfere with endogenous Arp2/3 complex regulatory pathways.

### SidG disrupts cytoskeletal architecture via Rac1- and Arp2/3-dependent mechanisms

**Fig 6:**
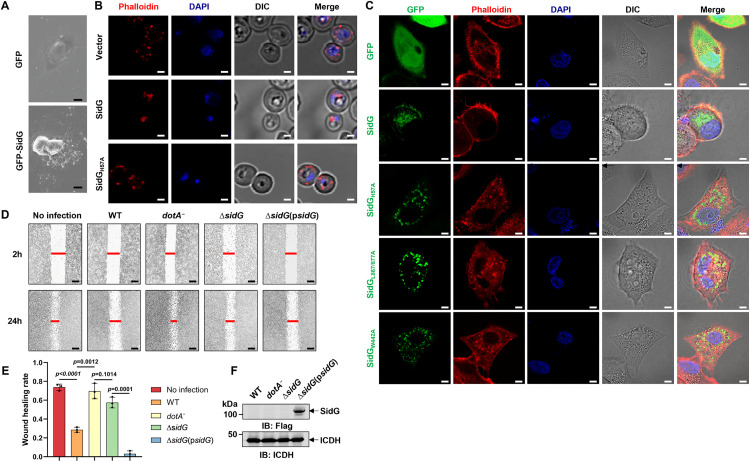
Cytoskeletal disruption by SidG requires the Cys-His-Asp triad, Rac1 engagement, and Arp2/3 targeting. **A** Representative SEM images of HEK293T cells expressing GFP or GFP-SidG. Images were taken at 24 h post-transfection. Scale bar, 2 μm. **B** Phalloidin staining (red) visualizing F-actin in W303 yeast strains expressing Flag-SidG or the H57A mutant. Nuclei were stained with DAPI (blue), and cell morphology was visualized by DIC. Protein expression in these yeast strains was induced with galactose for 6 **h.** Scale bar, 2 μm. **C** Immunofluorescence analysis of HeLa cells at 24 h after transfection with GFP-SidG or the H57A, L867A/L877A, and W442A mutants. F-actin was stained with phalloidin (red), nuclei were stained with DAPI (blue), and cell boundaries were visualized by DIC. Scale bar, 3 μm. **D**, **E** Wound healing assay of RAW 264.7 macrophages. Cells were infected for 2 h at an MOI of 50 with the indicated *L. pneumophila* strains. After a wound was created, representative images were taken at 2 h and 24 h post-wounding **(D)**, and the wound healing rate was quantified **(E)**. Data are mean ± SD (n = 3) and analyzed by one-way ANOVA. **F** Expression of SidG in the *L. pneumophila* strains was analyzed by immunoblotting with anti-Flag antibodies. ICDH served as a loading control. Data shown in **F** are representative from three independent experiments.

Current evidence indicates that the Arp2/3 complex represents the only known cellular nucleator capable of generating branched actin networks [22]. Thus, the observed interaction between SidG and Arp2/3 complex suggested that SidG might disrupt cytoskeletal architecture of host cells. Indeed, electron microscopy analysis demonstrated remarkable cellular shrinkage and morphological rounding in SidG-expressing mammalian cells **(**Fig 6A). Furthermore, phalloidin staining of yeast cells showed distinct cytoskeletal aggregates specifically in wild-type SidG-expressing cells, but not in those expressing the functionally inactive SidG_H57A_ mutant (Fig 6B). These cytoskeletal abnormalities likely underlie the observed growth inhibition phenotype. To systematically dissect the functional mechanisms of cytoskeletal disruption by SidG, we engineered three distinct loss-of-function variants: H57A (Cys-His-Asp triad inactivation), L867A/L877A (Rac1 binding ablation), and W442A (Arp2/3 engagement disruption). HeLa cells expressing these mutants all exhibited normal cytoskeletal structure by phalloidin staining (Fig 6C), revealing that all three domains are indispensable for cytoskeletal destabilization mediated by SidG. Furthermore, co-immunoprecipitation analysis demonstrated that the A domain is not necessary for SidG-Rac1 interaction (S9 Fig). Collectively, these results support our proposal that Rac1 functions as an allosteric activator for SidG, which in turn acts upon its downstream target, the Arp2/3 complex.

As the Arp2/3 complex orchestrates fundamental cellular processes, particularly cell migration, we examined the impact of SidG on cell motility by ectopic expression of SidG in host cells. Wild-type SidG, but not functionally inactive SidG_H57A_ mutant, potently suppressed cellular motility without compromising cell viability (S10 Fig). We further determined whether the observed phenotype by SidG occurs under physiological conditions and found that wild-type bacteria efficiently inhibited cell migration while a mutant missing SidG (Δ*sidG*) lost the ability to affect host cell motility during *L. pneumophila* infection (Fig 6D-Fig 6D and 6F). Overexpression of SidG in a Δ*sidG* strain resulted in a more potent inhibition of cell migration compared to the wild-type strain (Fig 6D-F). Thus, SidG targets the Arp2/3 complex to disrupt cytoskeletal architecture and subvert motility programs.

### ArpC3 rescues the yeast toxicity of SidG

**Fig 7:**
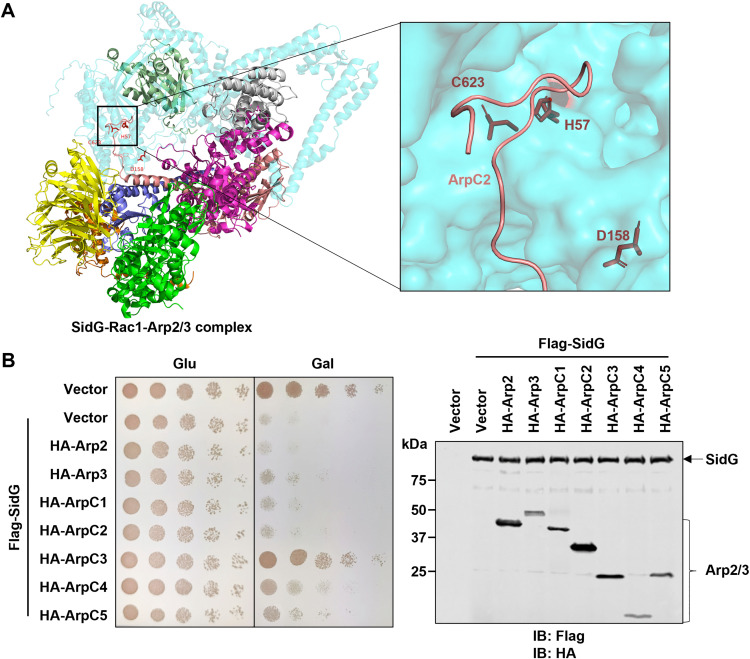
The Arp2/3 subunit ArpC3 suppresses the yeast toxicity of SidG. **A** AlphaFold-predicted structural model of SidG (cyan) in complex with Rac1 (olive green) and the Arp2/3 complex. The subunits are colored as follows: Arp2 (green), Arp3 (purple), ArpC1 (yellow), ArpC2 (red), ArpC3 (grey), ArpC4 (blue), and ArpC5 (orange). A magnified view of the predicted interface between SidG and ArpC2 with highlighted SidG Cys-His-Asp triad. **B** Yeast toxicity assay of SidG co-expressed with individual HA-tagged Arp2/3 subunits in W303. Protein expression was induced with galactose for 6 h and detected by immunoblotting with anti-Flag and anti-HA antibodies. Data shown in **B** are representative from three independent experiments.

A structural model of SidG-Arp2/3 revealed that the C-terminal of ArpC2 positions near the Cys-His-Asp pocket of SidG (Fig 7A), which raised the possibility that SidG may modify ArpC2. Unfortunately, we neither observed any shift bands of ArpC2 by co-expression of SidG (S11A Fig) nor detected any modification of ArpC2 mediated by SidG via mass spectrometric analysis. This discrepancy might suggest that the predicted proximity is coincidental, or it could reflect the inherent limitations in the accuracy of *in silico* structural predictions for large, multi-protein complexes. To further elucidate the mechanisms of cytoskeletal disruption by SidG via the Arp2/3 complex, we conducted a suppressor screening by individually co-expressing each of the seven Arp2/3 complex subunits in yeast expressing SidG. Strikingly, only ArpC3 overexpression completely rescued SidG-mediated yeast growth inhibition (Fig 7B). Despite extensive efforts, we were unable to detect SidG-induced modification of ArpC3 (S11B and S11C Fig). These findings suggest that SidG may modulate Arp2/3 through a non-covalent mechanism, potentially exploiting ArpC3 subunit as the critical regulatory node akin to the mechanisms mediated by WASF-family NPFs and Arpin.

### *L.* *pneumophila* employs SidG to enhance invasion

**Fig 8:**
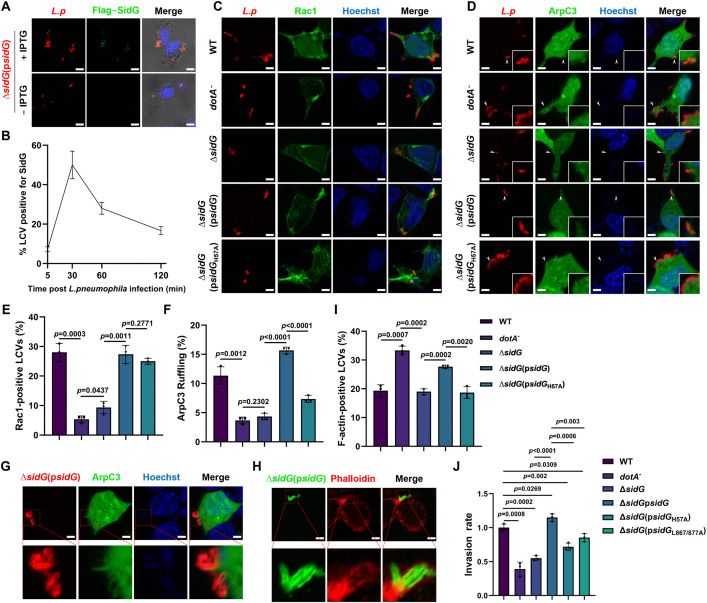
SidG facilitates host cell invasion in the initial phase of bacterial infection. **A**, **B** SidG is associated with the LCV during bacterial infection. HEK293 cells expressing FcγRII were infected for the indicated times with *L. pneumophila* strains expressing IPTG-inducible Flag-SidG. Immunofluorescence analysis was performed using anti-Flag (green) and anti-*L. pneumophila* (red) antibodies. Representative images are shown in (**A**) and percentage of the SidG-positive vacuoles was quantified for at least 100 LCVs **(B)**. Scale bar, 10 μm. **C**, **E** GFP-Rac1 localization in *L. pneumophila* infected cells. HEK293 cells expressing GFP-Rac1 were infected with the indicated bacterial strains for 30 min at an MOI of 20. Immunofluorescence was performed with anti-*L. pneumophila* (red) antibodies, and the nuclei were stained with Hoechst (blue). Representative images are shown in (**C**) and percentage of the Rac1-positive LCVs was quantified (**E**) for at least 100 vacuoles. Scale bar, 4 μm. **D**, **F**, **G** Membrane ruffling visualized by GFP-ArpC3 recruitment. HEK293 cells expressing GFP-ArpC3 were infected for 30 min at an MOI of 20 with the indicated *L. pneumophila* strains. Bacteria were labeled in red with anti-*L. pneumophila* antibodies, and the nuclei were stained with Hoechst (blue). Representative overview images are shown in (**D**) with a magnified view shown in **(G)**. The percentage of bacteria inducing ArpC3-positive membrane ruffling was quantified (**F**) from at least 100 bacteria. Scale bar, 4 μm. **H**, **I** Phalloidin staining of F-actin in HEK293 cells infected with the indicated strains for 10 min at an MOI of 20. Bacteria were immunostained with antibodies specific for *L. pneumophila* (red) and the nuclei were stained with Hoechst (blue). Representative images in (**H**) show the encasement of bacteria by F-actin, and the percentage of F-actin-positive LCVs from at least 100 vacuoles is quantified in **(I)**. Scale bars, 4 μm. **J** Invasion of RAW 264.7 cells by *L. pneumophila*. Cells were infected with indicated *L. pneumophila* strains at an MOI of 100. At 10 min post infection, intracellular bacteria were quantified by colony-forming unit enumeration. Data in **B**, **E**, **F**, **I**, and **J** are mean ± SD (n = 3). The color key for bacterial strains in the bar graphs is shown in (**I**) and applies to (**E**, **F**, and **J**). All quantitative data were analyzed by one-way ANOVA. Data shown in **A**, **C**, **D**, **G**, and **H** are representative from three independent experiments.

Next, we investigated the roles of SidG in the life cycle of *L. pneumophila* during infection. Previous studies identified SidG as a Dot/Icm substrate capable of interbacterial protein transfer [23], implying that this effector is expressed and competent for translocation during the infectious stationary phase. To validate this pioneer effector characteristic and examine its spatiotemporal regulation, we tracked SidG dynamics during the initial phase of infection using a SidG-overexpression strain. Consistent with a pre-formed effector primed for immediate action, SidG was rapidly recruited to the LCV. The proportion of SidG-positive vacuoles increased sharply following infection initiation, peaking at around 30 mins post-bacterial uptake (Fig 8A and 8B). Furthermore, LCVs containing wild-type, Δ*sidG*(p*SidG*) and Δ*sidG*(p*SidG*_H57A_) *L. pneumophila* efficiently recruited Rac1 to their surface in the early infection phase, while vacuoles harboring Δ*sidG* mutant showed significantly reduced recruitment of Rac1 (Fig 8C and 8E), which is consistent with our results that SidG binds to Rac1 independent of the SidG Cys-His-Asp motif. Despite only modest recruitment of ArpC3 to the LCVs (Fig 8D), SidG overexpression significantly enhanced Arp2/3-dependent membrane ruffling at *L. pneumophila* invasion sites (Fig 8F and 8G), along with the increased actin encasement of intracellular bacteria (Fig 8H and 8I). These findings collectively suggest that *L. pneumophila* actively promotes host cell invasion through SidG-mediated cytoskeletal rearrangements. To further assess the physiological significance of SidG in bacterial infection, we quantified intracellular *L. pneumophila* at 10 minutes post-infection. Importantly, the Δ*sidG* mutant strain exhibited significantly impaired cellular invasion compared to wild-type bacteria. Complementation with wild-type SidG, but not SidG_H57A_ or the Rac1-binding defective SidG_L867A/L877A_ mutant, restored the invasive capacity of the Δ*sidG* strain (Fig 8J). Consistently, further analysis revealed that SidG specifically accelerates the kinetics of bacterial internalization during the early stages of infection (S12 Fig).

We also examined the effect of SidG in intracellular replication of *L. pneumophila* by performing infection in bone marrow-derived macrophages (BMDMs) and the protozoan host *Dictyostelium discoideum*. Consistent with previous studies [24], the Δ*sidG* mutant grew at rates indistinguishable from those of the wild-type strain in both laboratory infection models (S13A and S13B Fig). Also, *sidG* deletion did not affect the proportion of replicative LCVs (S13C and S13D Fig), suggesting that SidG is not required for the formation of the replication vacuoles. Taken together, these results indicate that SidG promotes *L. pneumophila* invasion but is dispensable for its intracellular replication.

## Discussion

To successfully colonize host cells, intracellular bacterial pathogens must establish and maintain a stable replicative niche [25]. This process critically depends on their ability to manipulate the host cytoskeleton, particularly by hijacking actin dynamics to control membrane remodeling, vesicular trafficking, and niche stability [10]. Through evolutionary adaptation, bacteria have developed two principal mechanistic strategies to hijack host actin dynamics. The first strategy is to directly modulate actin functions by post-translational modifications. For example, the *Clostridium botulinum* C2 toxin and *Photorhabdus luminescens* Tc toxin affect actin polymerization by ADP-ribosylation [12,26]. The *Vibrio* and *Aeromonas* ACD toxin cross-links actin monomers and thus prevents actin polymerization [27]. Some pathogens impair the host cytoskeleton by direct proteolytic cleavage of actin, including grimelysin and protealysin from *Serratia* species and the *Legionella* effector RavK [28,29]*.* Another strategy involves targeting or mimicking host regulatory proteins that control actin dynamics. *Listeria monocytogenes* ActA and *Burkholderia* BimA exploit their WH2 domains to functionally imitate host nucleators, facilitating actin nucleation, elongation, and filament bundling [30,31]. Similarly, *Vibrio* VopF/VopL utilize tandem WH2 domains for dual activities in pointed-end actin elongation and nucleation [32], while *Rickettsia* Sca2 uniquely mimics formins by specifically promoting barbed-end elongation [33]. Alternatively, pathogens target host actin nucleators or regulatory proteins. *Shigella* IcsB and *Vibrio* MARTX toxins subvert Rho GTPases through N^ε^-fatty acylation [14,15], while *L. pneumophila* LegK2 phosphorylates the Arp3/ArpC1B subunits of the Arp2/3 complex [34]. In this study, our findings reveal SidG as a novel T4SS effector that uniquely integrates both Rac1-dependent signaling and Arp2/3 complex modulation to mediate host cytoskeleton remodeling during *L. pneumophila* infection.

While a recent study had identified the Cys-His-Asp triad in SidG [17], our integrated bioinformatic and functional analyses also defined key structural residues, such as W32 and W148, that are requisite for the triad-mediated function. Importantly, we further elucidated a molecular switch governing the function of SidG. We propose a multi-step model in which SidG is maintained in a quiescent state until its C-terminal domain engages the host GTPase Rac1. The association of Rac1 not only releases auto-inhibition by liberating the N-terminal domain but also likely induces an allosteric conformational change that stabilizes the functional configuration of the Cys-His-Asp triad. This explains why the truncated SidG_1-797_, despite being capable of binding the Arp2/3 complex, fails to exhibit toxicity to yeast. This host factor-dependent activation strategy mirrors the regulatory logic employed by other *Legionella* effectors, such as SidJ and VipD [35,36], which rely on host Calmodulin and Rab GTPases, respectively. Such a mechanism effectively licenses bacterial effector activity exclusively within the eukaryotic cytosol, imposing a strict spatiotemporal checkpoint to prevent premature activation. The activated conformation then engages the Arp2/3 complex with ArpC3 subunit as the key factor, as evidenced by genetic suppression of SidG-induced yeast toxicity upon ArpC3 overexpression. Crucially, while the Cys-His-Asp triad is biochemically essential, we observed no evidence of Arp2/3 component cleavage or modification. Therefore, we suggest that SidG modulates Arp2/3 complex activity through a mechanism that does not involve detectable covalent modification, potentially via allosteric modulation, with ArpC3 serving as the critical regulatory node in this pathogen-host interface. Furthermore, while SidG is lethal to yeast, it inhibits mammalian cell migration without compromising viability. We attribute this discrepancy to distinct physiological and temporal contexts: actin polymerization is indispensable for yeast division and proliferation, whereas SidG-mediated cytoskeletal disruption in mammalian cells specifically impairs motility while cellular metabolism remains intact during acute expression. This confirms that the observed migration defect is a direct consequence of cytoskeletal perturbation rather than a secondary effect of cell toxicity.

Our study establishes SidG as a distinctive addition to the extensive arsenal of cytoskeletal effectors employed by *L. pneumophila*, which also includes VipA, MavH, WipA, Ceg14, RavK, and LegK2 [29,34,37–40]. Although dispensable for intracellular bacterial replication, SidG’s distinct role in facilitating invasion, coupled with its rapid spatiotemporal recruitment to the LCV, defines it as a pioneer effector tailored for the nascent stages of infection. A key limitation of the current study remains the incomplete understanding of the precise mechanism mediated by the Cys-His-Asp triad. Future investigations dissecting the molecular basis of this motif will be essential to fully resolve this sophisticated strategy of SidG-mediated cytoskeletal subversion.

## Materials and methods

### Ethics statement

The animal study was reviewed and approved by the Institutional Animal Care and Use Committee of Jilin University (permit number: SY202412058).

### Plasmids and bacterial strains

The bacterial strains and plasmids used in this study are detailed in S1 and S2 Tables, respectively. Primers are listed in S3 Table. *Escherichia coli* strains were routinely cultured at 37°C in Luria-Bertani (LB) broth or on LB agar plates supplemented with appropriate antibiotics. All *L. pneumophila* strains used were derived from the parental strain Lp02 [41], and harbor the pZL507 plasmid to complement the *thyA* auxotrophy. *L. pneumophila* was cultured on CYE agar plates or in AYE broth. Both media were supplemented with 400 mg/L L-cysteine and 100 mg/L ferric nitrate. Liquid cultures were grown at 37°C with shaking at 220 rpm to the post-exponential phase (OD_600_ = 3.3-3.8) before use. The *sidG* deletion mutant (Lp02Δ*sidG*) was constructed using the suicide vector pSR47S via allelic exchange, following previously described methods [42]. Briefly, the upstream and downstream flanking regions of the *sidG* gene were amplified from Lp02 genomic DNA using specific primer pairs (S3 Table). These fragments were subsequently joined by overlap extension PCR to generate a deletion allele. The PCR product was digested with BamHI and SalI and ligated into the corresponding sites of pSR47S. For complementation experiments, the coding sequence of *sidG* was cloned into the vector pZL507. The resulting plasmid was introduced into the corresponding mutant strain by electroporation. For protein expression in mammalian cells, the relevant coding sequences were PCR-amplified and subcloned into expression vectors including pEGFP-C1, pCMV-HA, or pCMV-Flag.

### Cell culture and transfection

HeLa, HEK293, and HEK293T cell lines were obtained from the American Type Culture Collection (ATCC). Cells were maintained in Dulbecco’s modified Eagle’s medium (DMEM) supplemented with 10% (v/v) fetal bovine serum (FBS) at 37°C in a humidified incubator with 5% CO_2_. BMDMs were prepared from A/J mice as previously described [43]. All animal procedures were approved by the Institutional Animal Care and Use Committee of Jilin University (permit number: SY202412058). For transient expression of exogenous proteins, cells were seeded in 6-well plates and transfected at approximately 80% confluency. Per well, 2.5 μg of total plasmid DNA was delivered using 3.5 μL Lipofectamine 3000 reagent and 3.5 μL P3000 reagent (Invitrogen), following the manufacturer’s instructions.

### Recombinant protein expression and purification

Recombinant GST-tagged Rac1 and PAK1 PBD proteins were expressed in *E. coli* BL21 (DE3) cells. Transformed cells were initially grown overnight at 37°C in LB medium containing 100 µg/mL ampicillin. This culture was then diluted 1:200 into 1 L of fresh LB-ampicillin medium and cultivated at 37°C with shaking until reaching OD_600_ of 0.6-0.8. Protein expression was subsequently induced with 0.1 mM IPTG (isopropyl β-D-1-thiogalactopyranoside) and incubated for 16–18 h at 18°C with shaking. Cells were harvested by centrifugation at 4,000 × *g* for 20 minutes at 4°C. The resulting pellet was resuspended in ice-cold PBS supplemented with protease inhibitors and 1 mM DTT, and then lysed using a homogenizer (JN-mini, JNBIO, Guangzhou, China). Cell debris was removed by centrifugation at 12,000 × *g* for 30 minutes at 4°C to obtain the soluble lysate. The soluble fraction was incubated with Glutathione Agarose Resin (Sangon Biotech, Shanghai, China) for 2 h at 4°C with gentle rotation to facilitate binding. Bound GST-tagged proteins were eluted with 10 mM reduced glutathione (GSH). Finally, eluted fractions were pooled and subjected to two rounds of dialysis against storage buffer (50 mM Tris-HCl pH 8.0, 150 mM NaCl, 10% (v/v) glycerol, 1 mM DTT) at 4°C.

### Yeast manipulation and toxicity assays

The *Saccharomyces cerevisiae* strain used in this study was W303. Yeast strains were typically grown at 30°C in rich YPD medium (1% yeast extract, 2% peptone, 2% dextrose). For plasmid selection, transformants were grown on synthetic defined (SD) medium lacking the appropriate nutrient corresponding to the plasmid auxotrophic marker, specifically uracil for pSB157M or leucine for p425GPD. Yeast transformation was performed using the standard lithium acetate/single-stranded carrier DNA/PEG method [44].

The homozygous *RHO5* deletion strain was generated using a PCR-based homologous recombination strategy. A deletion cassette containing the *TRP1* marker flanked by 850 bp upstream and downstream homologous regions of *RHO5* was generated and transformed into W303 cells. Transformants were selected on SD medium lacking tryptophan (SD/-Trp), and the deletion was verified by sequencing and RT-qPCR. Primers used for strain construction and RT-qPCR are listed in S3 Table.

To assess toxicity, overnight cultures were serially diluted 5-fold, and 10 μL of each dilution was spotted onto SD plates containing either glucose or galactose. Plates were incubated at 30°C for 2 days and photographed. Yeast cell lysates were prepared for immunoblotting according to established protocols.

### RT-qPCR

Total RNA was extracted from yeast cells using TRIzol Reagent (Thermo Fisher Scientific) combined with acid-washed glass beads for mechanical lysis. cDNA was synthesized using the 5X All-In-One RT MasterMix (Abm). Quantitative PCR was performed using BlasTaq 2X qPCR MasterMix (Abm). Relative mRNA levels of *RHO5* were calculated using the 2^-ΔΔCt^ method, with PGK1 serving as the internal reference control.

### Immunoprecipitation and GST pull-down

HEK293T cells were transiently transfected with relevant plasmids. 24 h post-transfection, cells were harvested by washing once with ice-cold PBS and collected by centrifugation at 500 × *g* for 5 minutes at 4°C. Cell pellets were resuspended and lysed in ice-cold RIPA buffer supplemented with a protease inhibitor (Sigma) on ice for 10 minutes with occasional vortexing. Cell lysates were then clarified by centrifugation at 14,000 × *g* for 10 minutes at 4°C. The resulting supernatant was collected. For immunoprecipitation, the resulting supernatant was incubated with 10 µL slurry of anti-HA, anti-Flag, or anti-GFP agarose beads (AlpalifeBio) overnight at 4°C with gentle end-over-end rotation. Following incubation, the beads were washed 3 times with ice-cold RIPA buffer. Bound proteins were eluted from the beads by adding 30 µL of 1 × SDS-PAGE loading buffer and boiling at 95°C for 5 minutes. Eluted samples, along with input controls (40 µL of the initial lysate), were resolved by SDS-PAGE.

For the pull-down assay, 50 µg of purified GST-fusion protein was pre-incubated with 100 µL slurry of Glutathione High Capacity Magnetic Agarose Beads (Sigma) for 2 h at 4°C with gentle rotation to allow binding. After incubation, the beads were washed 3 times to remove unbound GST protein. Subsequently, the GST-protein-bound beads were incubated with the prey protein source. For the direct interaction assay shown in Fig 2D, a co-expression strategy was used to circumvent challenges with His_6_-SidG purification. Lysate from *E. coli* co-expressing GST-Rac1 and His_6_-SidG was incubated with glutathione beads overnight at 4°C. Finally, bound proteins were eluted by adding 30 µL of 1 × SDS-PAGE loading buffer and boiling at 95°C for 5 minutes. Eluted samples were analyzed by SDS-PAGE and immunoblotting.

For the interaction assay between SidG and individual Arp2/3 subunits, a lysate-based co-immunoprecipitation strategy was employed. Bacterial lysates were prepared using bacterial active protein extraction reagent (Beyotime). To ensure detection of potential interactions independent of Rac1-mediated activation, bacterial lysates expressing GST-SidG were mixed with those expressing the Rac1-independent mutant GST-SidG_1-797_ to serve as the bait pool. This mixture was incubated with lysates containing individual His-Flag-tagged Arp2/3 subunits or His-Flag-Rac1. Immunoprecipitation was performed using Anti-Flag Affinity Gel (Sigma) at 4°C for 4 h. After washing three times with lysis buffer, bound proteins were eluted and analyzed by immunoblotting.

### Antibodies and immunoblotting

For immunoblotting analysis, protein samples were resolved by SDS-PAGE and transferred onto nitrocellulose membranes (Pall Life Sciences). Membranes were blocked with 5% non-fat dry milk in PBS containing 0.05% Tween-20 (PBS-T) for 1 h at room temperature. Subsequently, membranes were incubated overnight at 4°C with primary antibodies. Following washes with PBS-T, membranes were incubated with appropriate infrared dye-conjugated secondary antibodies for 1 h at room temperature. After final washes in PBS-T, membranes were imaged and fluorescence signals were acquired using the Odyssey CLx Infrared Imaging System (LI-COR Biosciences). A detailed list of primary antibodies and their working dilutions used in this study is provided in S4 Table.

### Rac1 activation assay

Rac1 activation was assessed using a GST-PBD pull-down assay. Briefly, HEK293T cells were transfected with either HA-Rac1 or GFP-SidG, GFP-SidG_H57A_ mutant, or the GFP control. After 24 h, cells were harvested and lysed separately in ice-cold RIPA buffer. Lysates were clarified by centrifugation at 14,000 × *g* for 10 min at 4°C. Subsequently, lysates containing HA-Rac1 were combined with lysates containing either GFP-SidG, GFP-SidG_H57A_ mutant, or the GFP control, and incubated at 37°C for 1 h. Supernatants were incubated with purified GST-PAK1 PBD immobilized on glutathione resin for 40 minutes at 4°C with gentle mixing. The beads were washed three times with RIPA buffer, and bound proteins (activated Rac1) were eluted by boiling in SDS sample loading buffer. Eluted proteins were resolved by SDS-PAGE and detected by Western blot analysis.

### Random mutagenesis by hydroxylamine

Random mutagenesis of the p425GPD-SidG plasmid was performed by hydroxylamine treatment, following a previously described protocol [45]. In brief, 5 µg of plasmid DNA was incubated in a 100 µL reaction mixture containing 0.5 M hydroxylamine, 5 mM Tris-HCl (pH 6.0), and 0.5 mM Na_2_-EDTA. The reaction was carried out for 10 h at 37°C or alternatively for 1 h at 65°C. Following the incubation, the mutagenized DNA product was recovered and transformed into the W303 for subsequent analysis.

### Immunofluorescence staining

HeLa or HEK293 cells were seeded onto sterile glass coverslips placed in 24-well, transfected with the indicated plasmids. At 24 h post-transfection, cells were washed once with PBS and fixed with 4% paraformaldehyde (PFA) for 30 minutes at room temperature (RT). Following fixation, cells were permeabilized with 0.2% Triton X-100 in PBS for 5 minutes, then blocked with 4% goat serum (Beyotime) for 30 minutes at RT. Cells were incubated with primary antibodies for 1 h at RT or overnight at 4°C. After washing with PBST, cells were incubated for 1 h at RT (protected from light) with appropriate secondary antibodies: Texas Red-conjugated goat anti-Mouse IgG (Thermo Fisher Scientific, T6390, 1:500) or Alexa Fluor 488-conjugated Goat anti-Rabbit IgG (Thermo Fisher Scientific, A-11008, 1:500). Nuclei were counterstained with DAPI or Hoechst 33342 (Beyotime, C1022, 1:10000). Images were acquired on an Olympus FV3000 confocal microscope.

### Differential staining for invasion kinetics

To distinguish between extracellular and intracellular bacteria during the early stages of infection (0–2 h), a differential staining protocol was employed. Cells were infected with the indicated *L. pneumophila* strains and fixed with 4% paraformaldehyde at the indicated time points. To label extracellular bacteria, cells were incubated with a rat anti-*L. pneumophila* antibody, followed by staining with an Alexa Fluor 488-conjugated anti-rat secondary antibody (Abcam) prior to permeabilization. Subsequently, cells were permeabilized with 0.2% Triton X-100. Total bacteria (both extracellular and intracellular) were then detected by incubation with a rabbit anti-*L. pneumophila* antibody, followed by an Alexa Fluor 647-conjugated anti-rabbit secondary antibody (Abcam).

### Infection and replication assays

For intracellular replication assays, BMDMs (4 × 10⁵ cells/well) or *Dictyostelium discoideum* (5 × 10⁵ cells/well) were seeded into 24-well plates and allowed to adhere overnight. *L. pneumophila* strains were used to infect the host cells at a multiplicity of infection (MOI) of 0.05 for BMDMs (incubated at 37°C) or 0.1 for *D. discoideum* (incubated at 25°C). After 2 h of infection, extracellular bacteria were removed by washing wells three times with PBS and incubating the cells with medium containing 100 µg/mL gentamicin for 1 h to kill any remaining extracellular bacteria. Cells were collected and lysed using 0.02% saponin in sterile PBS at the indicated time points. Lysates were serially diluted and plated onto CYE agar plates. Colony Forming Units (CFUs) were enumerated after incubation at 37°C for 3–4 days to determine intracellular bacterial numbers.

For immunofluorescence analysis of infected cells, HEK293 cells were seeded onto sterile glass coverslips placed in 24-well plates. Cells were co-transfected with plasmids encoding GFP-Rac1/ArpC3 along with plasmid pJC119R, which express FCγRII. At 24 h post-transfection, bacteria were opsonized with anti-*L. pneumophila* antibody for 30 min at 37°C prior to infecting the cells at an MOI of 20. Following infection, cells were processed for immunofluorescence as described in the ‘Immunofluorescence Staining’ section.

To quantify bacterial invasion, RAW 264.7 cells were seeded into 24-well plates at a density of 4 × 10⁵ cells/well and allowed to adhere overnight. *L. pneumophila* strains, grown to post-exponential phase, were used to infect the cells at an MOI of 100. The infection was synchronized by centrifuging the plates at 1,000 × *g* for 5 min. After a 10-minute incubation at 37°C, cells were washed and treated with gentamicin as described above to eliminate extracellular bacteria. The number of invaded bacteria was then determined by enumerating CFUs following cell lysis, as described for the intracellular replication assay.

### Mass spectrometry analysis

To identify proteins interacting with SidG constructs, immunoprecipitated samples were resolved by SDS-PAGE. Protein bands were visualized by Coomassie Brilliant Blue staining, excised from the gel, and subjected to in-gel digestion with trypsin. The resulting peptide mixtures were analyzed by LC-MS/MS using an Orbitrap Eclipse Tribrid mass spectrometer (Thermo Fisher Scientific).

### Wound healing assay

The wound healing assay was performed following previously described methods [46]. Briefly, HEK293 or RAW 264.7 cells were seeded into 6-well plates and allowed to reach approximately 90% confluence, forming a cell monolayer. A linear scratch, simulating a wound, was created in the center of the monolayer using a sterile 200 µL pipette tip. Detached cells and debris were then removed by gently washing the plates with serum-free medium. Following the wash, the cells were incubated at 37°C. Images of the scratch area, representing the initial wound width (2 h time point), were captured using an Olympus IX-83 microscope after 2 h of incubation. The cells were subsequently incubated for an additional 22 h. After the 24 h incubation period, images of the same scratch areas were acquired again to measure the final wound width (24 h time point). Wound healing was quantified using ImageJ software (https://imagej.net/ij/). The cell-free gap width was measured at multiple points along each scratch at both the 2 h (initial width) and 24 h (final width) time points. The percentage of wound closure was calculated using the following formula: % Wound Closure = [(Initial Width − Final Width)/ Initial Width] × 100.

### Cell viability assay

HEK293T cells were seeded in 96-well plates and transfected with the indicated plasmids. At 24 h post-transfection, 10 µL of Cell Counting Kit-8 solution (APExBIO) was added to each well containing 100 µL of medium. The plates were incubated at 37°C for 1–2 h, and the absorbance was measured at 450 nm using a multimode microplate reader (BioTek Synergy H1).

### Scanning electron microscopy (SEM)

HEK293T cells were cultured on sterile glass coverslips and transfected with plasmids encoding either GFP or GFP-SidG. Following transfection, cells were fixed with 2.5% glutaraldehyde at room temperature. After washing three times with PBS, samples were dehydrated through a graded ethanol series (30%, 50%, 70%, 80%, 90%, 95%, and 100% ethanol, 10 min each step). Samples were then allowed to air dry completely for at least 2 h. Dried coverslips were mounted onto aluminum stubs using conductive adhesive and sputter-coated with gold-palladium (Au-Pd). Cell surface morphology was examined using a Hitachi S-4800 scanning electron microscope at an accelerating voltage of 10 kV.

### Bioinformatics and structure prediction

Multiple sequence alignments, comparing SidG with homologs from different *Legionella* species, and relevant host proteins were generated using the CLUSTALW algorithm (https://www.genome.jp/tools-bin/clustalw). Conserved residues and motifs, including the Cys-His-Asp motif, were visualized and annotated using GeneDoc. HMMER (https://www.ebi.ac.uk/Tools/hmmer/search/phmmer) was used with profile Hidden Markov Models to analyze SidG for evolutionary sequence conservation and identify key conserved sequence features [47].

AlphaFold (https://alphafoldserver.com/) was used to generate *in silico* predictions of structural coordinates for SidG, its homologs, Rac1, and Arp2/3 complex components. The predicted three-dimensional structures were then visualized, superimposed, and examined using PyMOL (The PyMOL Molecular Graphics System, Version 2.5.5 Schrödinger, LLC.). Putative protein-protein interaction interfaces, particularly between SidG and Rac1, were investigated based on the predicted structures. Detailed analysis of potential interfacial residues, including predicted hydrogen bonds and hydrophobic contacts, was performed and visualized in two dimensions using LigPlot^+^ v.2.2. Schematic diagrams summarizing interaction contacts and protein secondary structure topology were generated using PDBsum (www.ebi.ac.uk/thornton-srv/databases/pdbsum/).

### Statistical analyses

Statistical significance between two groups was determined using the unpaired two-tailed Student’s t-test, while one-way ANOVA was used for multiple group comparisons. *p-*values less than 0.05 were considered statistically significant. Statistical analyses were performed using GraphPad Prism (GraphPad Software, La Jolla, CA, USA).

## Supporting information

S1 Fig Conservation analysis of SidG. Variability score plot of the amino acid sequence of *L. pneumophila* SidG.
(TIF)

S2 Fig The Cys-His-Asp triad is conserved in SidG homologs. (A) Sequence alignment of the Cys-His-Asp motif within SidG homologs. (B) Structural alignment of predicted models for SidG homologs. (C) Yeast toxicity assay of SidG orthologs from *LP*, *LB*, and *LS*. Protein expression was induced with galactose for 6 h and detected by immunoblotting with anti-Flag and anti-PGK1 antibodies. (D) Yeast toxicity assay of SidG_LS_ and its Cys-His-Asp triad mutants. Data shown in (C) and (D) are representative from three independent experiments.
(TIF)

S3 Fig Identification of SidG-Rac GTPase interaction. (A) IP-MS analysis using Flag-SidG_H57A_ as bait identified endogenous Rac1 and Rac3 as binding partners from HEK293T cell lysates. Sequence coverage for the identified Rac1 peptides is shown below. (B) Co-immunoprecipitation of HA-tagged Rac1 variants (WT, Q61L, T17N) with GFP-SidG_H57A_ in transfected HEK293T cells. Lysates were subjected to anti-HA immunoprecipitation followed by immunoblotting with anti-GFP and anti-HA antibodies. (C) Quantification of the band intensities shown in (B). Data are mean ± SD (n = 3) and analyzed by the unpaired two-tailed Student’s t-test. Data shown in (B) are representative of three independent experiments.
(TIF)

S4 Fig Rac1 expression fails to counteract the yeast toxicity of SidG. (A) Yeast toxicity assay of SidG co-expressed with the indicated HA-tagged Rac1 or its variants (Q61L and T17N). Protein expression was induced with galactose for 6 h. Lower panels show immunoblots probed with anti-Flag and anti-HA antibodies; PGK1 served as a loading control. (B) Lysates of HEK293T cells expressing HA-Rac1 were mixed with lysates from cells expressing GFP, GFP-SidG, or GFP-SidG_H57A_. The mixed lysates were then subjected to GST-PAK1 PBD pull-down assay. The bead-bound proteins were analyzed by immunoblotting with antibodies specific for GFP, HA, and GST. Data shown in (A) and (B) are representative of three independent experiments.
(TIF)

S5 Fig Analysis of the minimal region of SidG for its interaction with Rac1. (A) Two orthogonal views of the AlphaFold-predicted structural model of the SidG-Rac1 interaction complex. (B) Co-immunoprecipitation analysis to map the minimal Rac1-binding region of SidG. Lysates from HEK293T cells co-transfected with HA-Rac1 and the indicated GFP-tagged SidG truncation mutants were subjected to anti-HA immunoprecipitation and immunoblotting with anti-GFP and anti-HA antibodies. A schematic diagram of the SidG constructs is shown on the right. Data shown in (B) are representative of three independent experiments.
(TIF)

S6 Fig Identification of key residues mediating the SidG-Rac1 interaction. (A) LigPlot^+^ diagram of the predicted interaction interface between SidG and Rac1. (B, C) Co-immunoprecipitation analysis of HEK293T cells co-expressing HA-Rac1 and the indicated GFP-tagged SidG mutants. Lysates were subjected to anti-HA immunoprecipitation. (D) PDBsum diagrams showing the secondary structure of wild-type SidG and the L867A/L877A mutant. Data shown in (B) and (C) are representative of three independent experiments.
(TIF)

S7 Fig The yeast Rac1 homolog Rho5 is essential for SidG-induced toxicity. (A) Superposition of AlphaFold models of human Rac1 (blue) and *S. cerevisiae* Rho5 (orange). The C-terminal disordered region of Rho5_234–332_ was excluded. (B) Schematic of the *RHO5* deletion strategy. The coding sequence of Rho5 was replaced with the TRP1 marker. (C) RT-qPCR validation of the *RHO5* deletion strain. Relative mRNA levels were normalized to PGK1. Data are mean ± SD (n = 3, unpaired two-tailed Student’s t-test). (D) Rescue of SidG toxicity by human Rac1. The *rho5*∆::*TRP1* strain expressing Flag-SidG alone or co-expressing HA-Rac1 was spotted onto glucose or galactose plates. Lower panels: Immunoblots for indicated proteins.
(TIF)

S8 Fig SidG interaction relies on the integrity of the Arp2/3 complex. Lysate-based co-immunoprecipitation analysis of SidG binding to individual Arp2/3 subunits. Bacterial lysates containing a bait mixture of GST-SidG and GST-SidG_1-797_ were incubated with lysates containing His-Flag-tagged Rac1 or individual Arp2/3 subunits. Immunoprecipitated proteins by anti-Flag beads were analyzed by immunoblotting with anti-GST and anti-Flag antibodies. Data shown are representative of three independent experiments.
(TIF)

S9 Fig SidG interacts independently with Rac1 and the Arp2/3 complex. Co-immunoprecipitation of HA-Rac1 with GFP-SidG_H57A_ or GFP-SidG_H57A ΔA domain_ from co-transfected HEK293T cells. Immunoprecipitation was performed with anti-HA antibodies followed by immunoblotting with anti-HA and anti-GFP antibodies. Data shown are representative of three independent experiments.
(TIF)

S10 Fig SidG hampers cellular migration via the Cys-His-Asp motif. (A) HEK293 cells were scratched 24 h after transfection with GFP, GFP-SidG, or GFP-SidG_H57A_. Images were taken at 2 and 24 h post-scratch. Scale bar, 100 μm. (B) Quantification of the wound healing rate. Data are mean ± SD (n = 3) and analyzed by the unpaired two-tailed Student’s t-test. (C) Expression of GFP-SidG was detected by immunoblotting with anti-GFP antibodies. Actin served as a loading control. (D) Cell viability of HEK293T cells expressing the indicated proteins was assessed by CCK-8 assay at 24 h post-transfection. Data are mean ± SD (n = 3). ns, not significant. Data shown are representative of three independent experiments.
(TIF)

S11 Fig No modification of ArpC2 or ArpC3 by SidG was detected. (A, B) Lysates from cells transfected with GFP-SidG were mixed with lysates prepared from cells expressing Flag-ArpC2 (A) or Flag-ArpC3 (B) and incubated at 30°C for 6 h. Samples were resolved by SDS-PAGE under non-boiled or boiled conditions and analyzed by immunoblotting. Right panels showed detection of GFP-SidG expression after sequential immunoprecipitation (anti-Flag, then anti-GFP). (C) Yeast cells co-expressing HA-ArpC3 and either GFP or GFP-SidG were induced with galactose overnight. Lysates were subjected to anti-HA immunoprecipitation followed by immunoblotting with anti-HA and anti-GFP antibodies (left) or Coomassie Brilliant Blue staining (right). Data shown are representative of three independent experiments.
(TIF)

S12 Fig SidG facilitates rapid internalization of *L. pneumophila* during early infection. (A) Representative images of differential staining distinguishing extracellular (green) and total (magenta) bacteria. Scale bar, 2 μm. (B) Quantification of intracellular bacteria per cell over time in RAW 264.7 cells infected with WT or ∆*sidG* strains (MOI = 50). Data are mean ± SD (n = 3, at least 50 cells quantified per replicate) and analyzed by the unpaired two-tailed Student’s t-test.
(TIF)

S13 Fig SidG is not essential for intracellular growth of *L. pneumophila.* (A, B) Intracellular growth curves of the indicated *L. pneumophila* strains were performed in BMDMs (A) and *D. discoideum* (B). (C) Representative immunofluorescence images of BMDMs infected with the indicated *L. pneumophila* strains for 12 h at an MOI of 10. Bacteria were stained with the anti-*L. pneumophila* antibody (green). Nuclei were stained with Hoechst (blue). Scale bar, 2 μm. (D) Quantification of replicative vacuoles from the experiment in (C) at 12 h post-infection. Data are mean ± SD (n = 3, > 100 LCVs per condition per experiment). Uncropped scans of Western blots. (Uncropped scans of Western blots.pdf).
(TIF)

S1 Table Bacterial and yeast strains used in this study. (DOCX)

S2 Table Plasmids used in this study. (DOCX)

S3 Table Primers used in this study. (DOCX)

S4 Table Antibodies used in this study. (DOCX)
