# Supplementary figures and images for: Legionella effector protein SidG disrupts host cytoskeleton via targeting Arp2/3 complex
Source: PLoS Pathog. 2026 Feb 9;22(2):e1013957. doi: 10.1371/journal.ppat.1013957 (PMC12904589; doi:10.1371/journal.ppat.1013957)

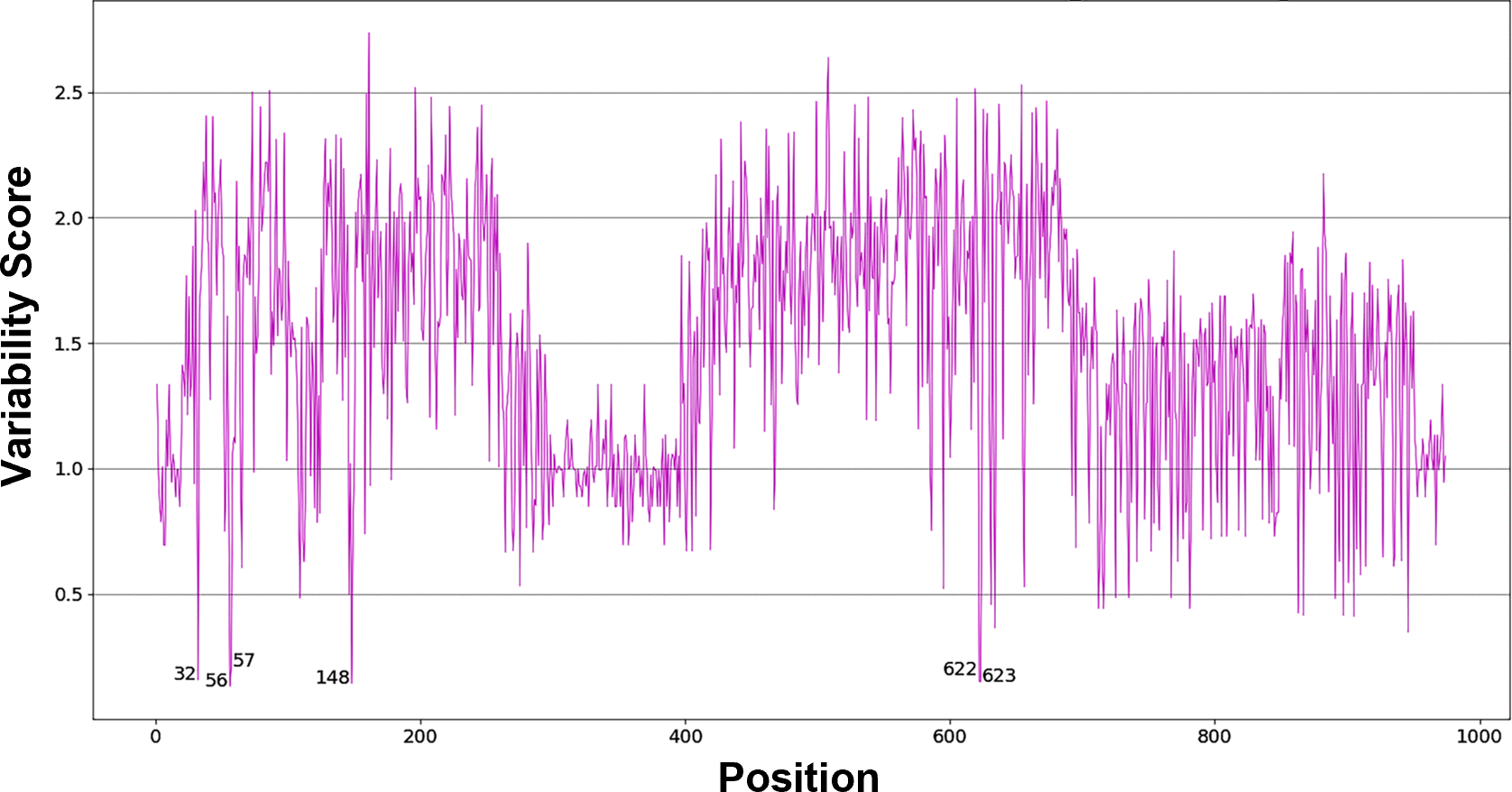

Supplement: S1 Fig — Variability score plot of the amino acid sequence of L. pneumophila SidG. (TIF) [file ppat.1013957.s001.tif]

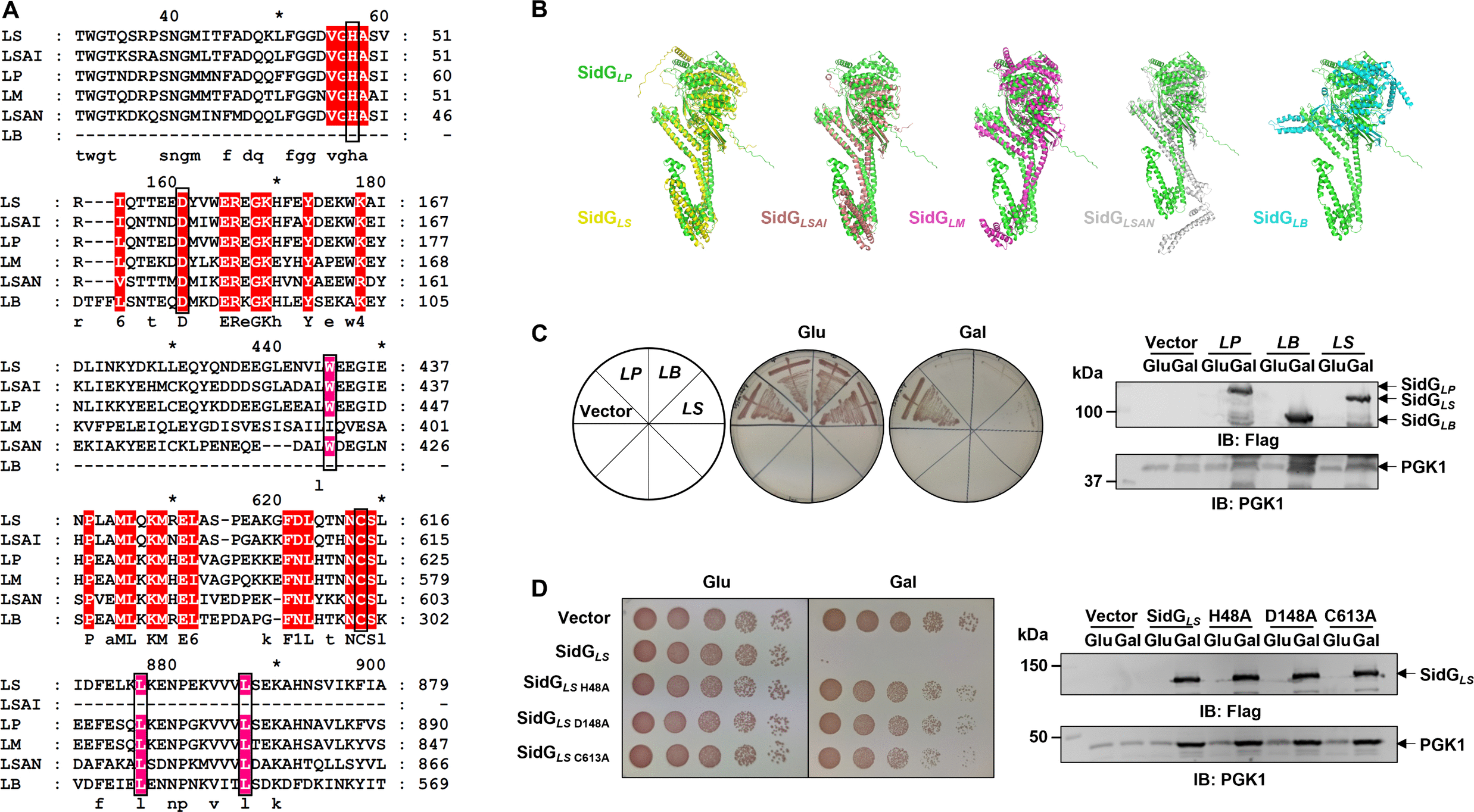

Supplement: S2 Fig — (A) Sequence alignment of the Cys-His-Asp motif within SidG homologs. (B) Structural alignment of predicted models for SidG homologs. (C) Yeast toxicity assay of SidG orthologs from LP, LB, and LS. Protein expression was induced with galactose for 6 h and detected by immunoblotting with anti-Flag and anti-PGK1 antibodies. (D) Yeast toxicity assay of SidGLS and its Cys-His-Asp triad mutants. Data shown in (C) and (D) are representative from three independent experiments. (TIF) [file ppat.1013957.s002.tif]

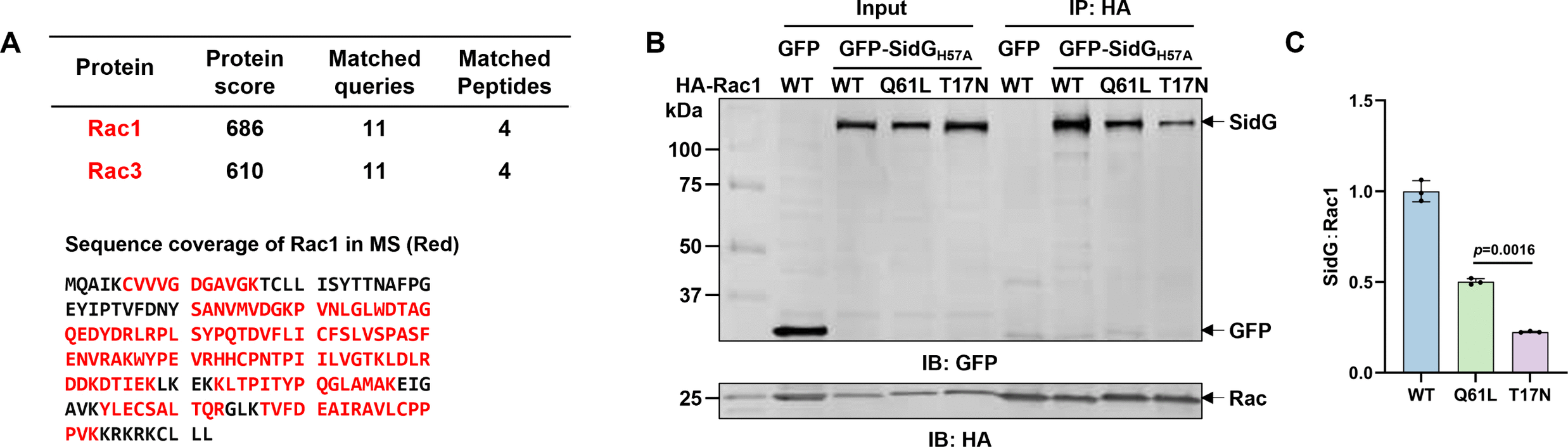

Supplement: S3 Fig — (A) IP-MS analysis using Flag-SidGH57A as bait identified endogenous Rac1 and Rac3 as binding partners from HEK293T cell lysates. Sequence coverage for the identified Rac1 peptides is shown below. (B) Co-immunoprecipitation of HA-tagged Rac1 variants (WT, Q61L, T17N) with GFP-SidGH57A in transfected HEK293T cells. Lysates were subjected to anti-HA immunoprecipitation followed by immunoblotting with anti-GFP and anti-HA antibodies. (C) Quantification of the band intensities shown in (B). Data are mean ± SD (n = 3) and analyzed by the unpaired two-tailed Student’s t-test. Data shown in (B) are representative of three independent experiments. (TIF) [file ppat.1013957.s003.tif]

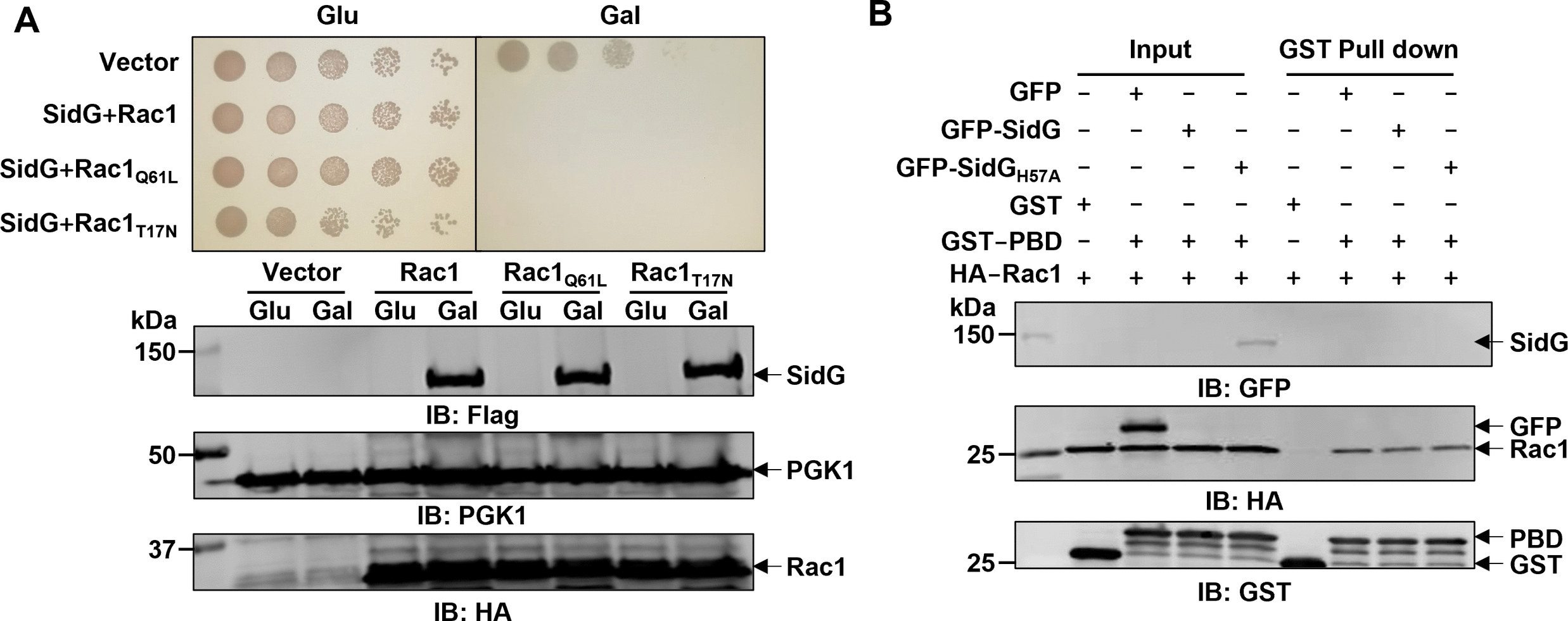

Supplement: S4 Fig — (A) Yeast toxicity assay of SidG co-expressed with the indicated HA-tagged Rac1 or its variants (Q61L and T17N). Protein expression was induced with galactose for 6 h. Lower panels show immunoblots probed with anti-Flag and anti-HA antibodies; PGK1 served as a loading control. (B) Lysates of HEK293T cells expressing HA-Rac1 were mixed with lysates from cells expressing GFP, GFP-SidG, or GFP-SidGH57A. The mixed lysates were then subjected to GST-PAK1 PBD pull-down assay. The bead-bound proteins were analyzed by immunoblotting with antibodies specific for GFP, HA, and GST. Data shown in (A) and (B) are representative of three independent experiments. (TIF) [file ppat.1013957.s004.tif]

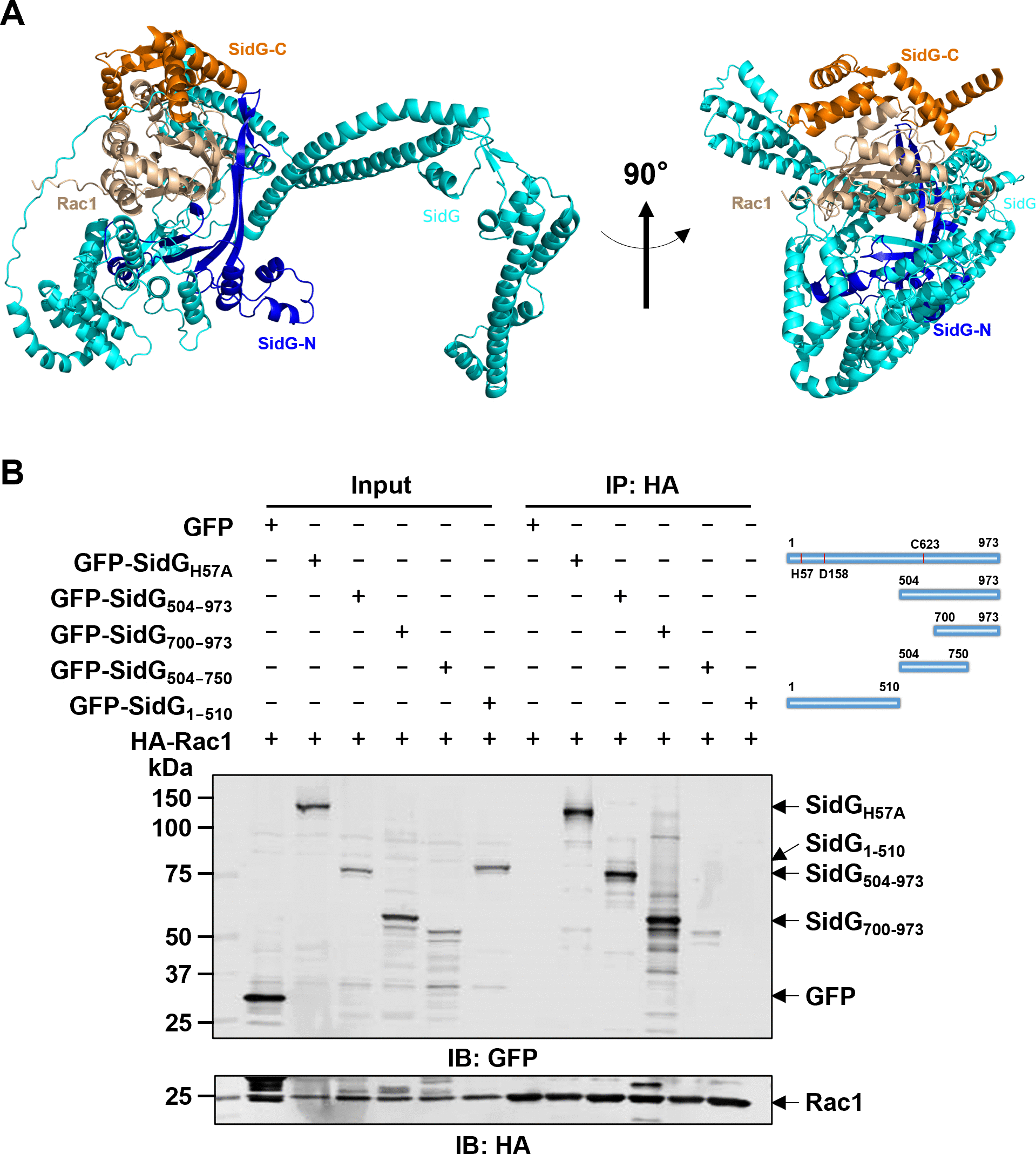

Supplement: S5 Fig — (A) Two orthogonal views of the AlphaFold-predicted structural model of the SidG-Rac1 interaction complex. (B) Co-immunoprecipitation analysis to map the minimal Rac1-binding region of SidG. Lysates from HEK293T cells co-transfected with HA-Rac1 and the indicated GFP-tagged SidG truncation mutants were subjected to anti-HA immunoprecipitation and immunoblotting with anti-GFP and anti-HA antibodies. A schematic diagram of the SidG constructs is shown on the right. Data shown in (B) are representative of three independent experiments. (TIF) [file ppat.1013957.s005.tif]

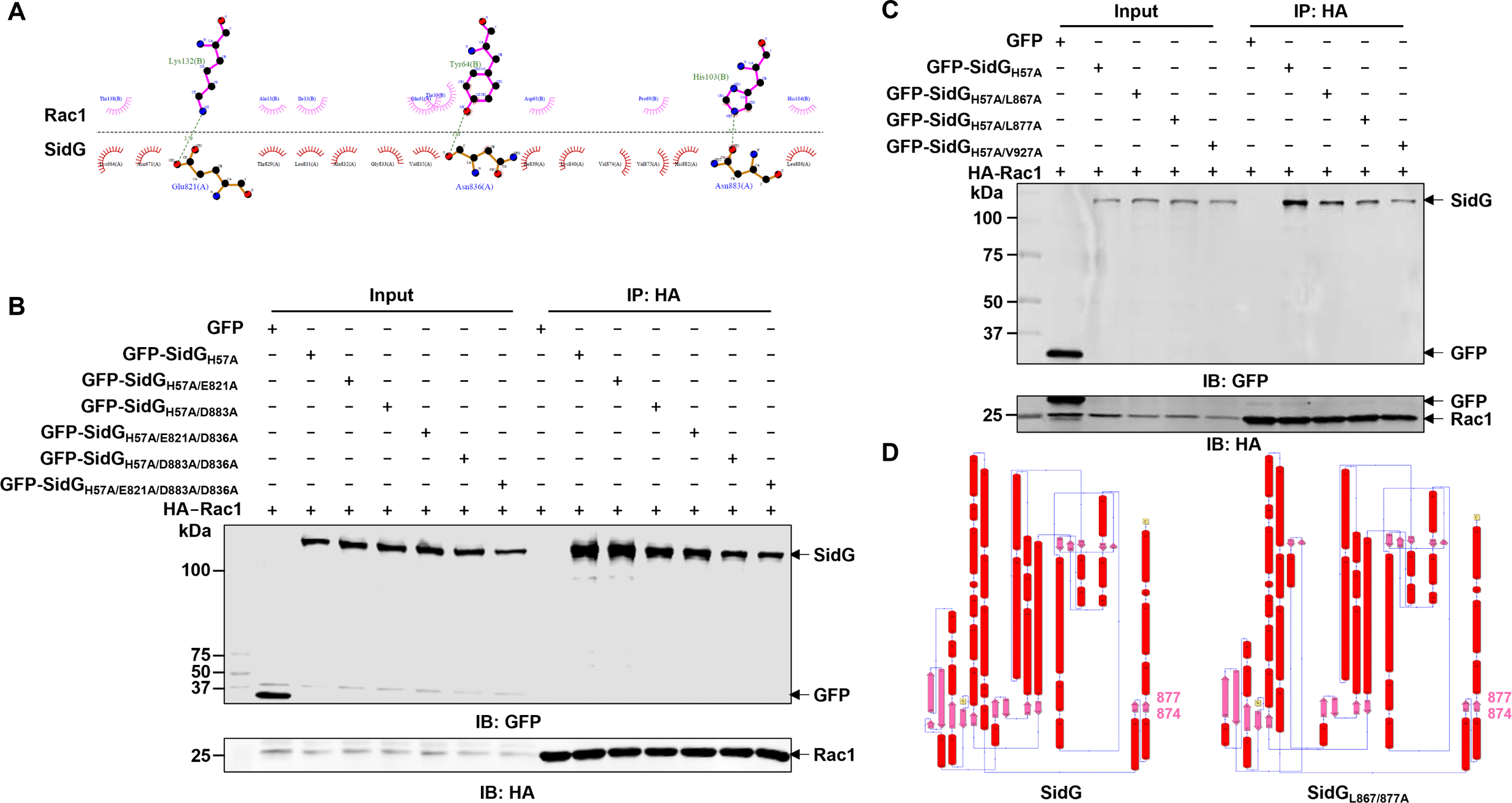

Supplement: S6 Fig — (A) LigPlot+ diagram of the predicted interaction interface between SidG and Rac1. (B, C) Co-immunoprecipitation analysis of HEK293T cells co-expressing HA-Rac1 and the indicated GFP-tagged SidG mutants. Lysates were subjected to anti-HA immunoprecipitation. (D) PDBsum diagrams showing the secondary structure of wild-type SidG and the L867A/L877A mutant. Data shown in (B) and (C) are representative of three independent experiments. (TIF) [file ppat.1013957.s006.tif]

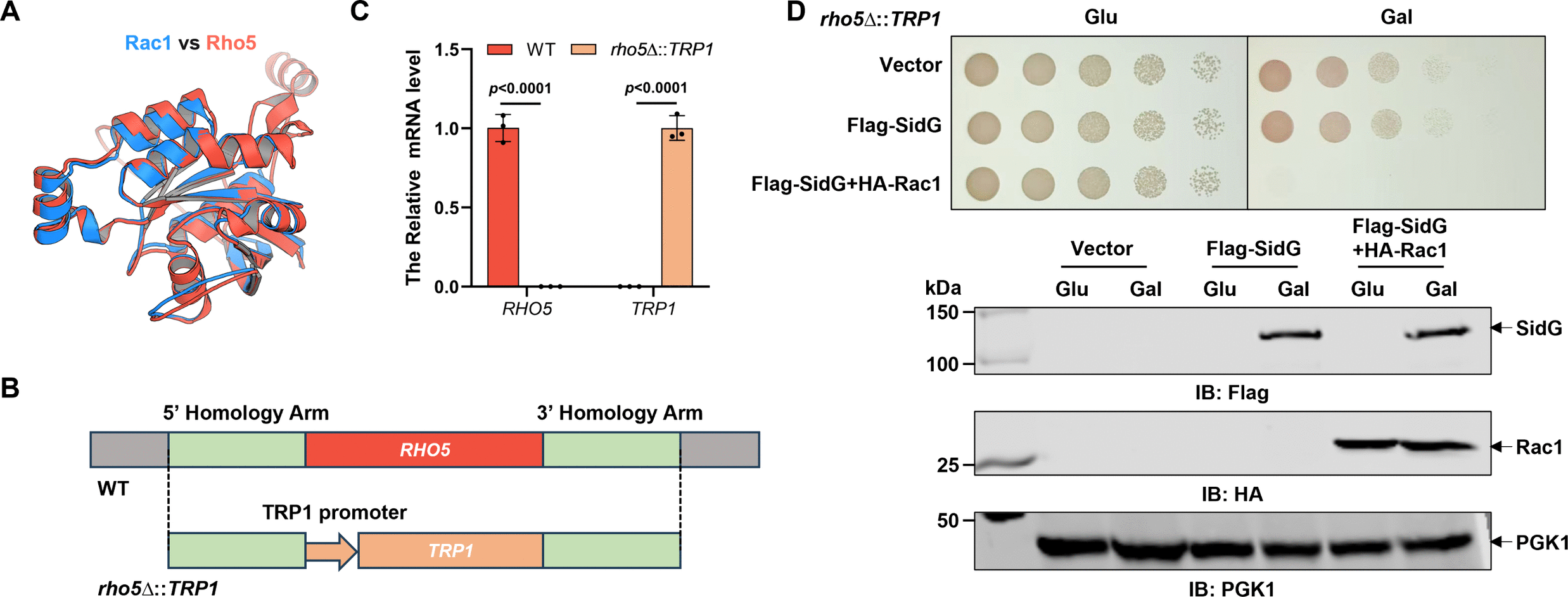

Supplement: S7 Fig — (A) Superposition of AlphaFold models of human Rac1 (blue) and S. cerevisiae Rho5 (orange). The C-terminal disordered region of Rho5234–332 was excluded. (B) Schematic of the RHO5 deletion strategy. The coding sequence of Rho5 was replaced with the TRP1 marker. (C) RT-qPCR validation of the RHO5 deletion strain. Relative mRNA levels were normalized to PGK1. Data are mean ± SD (n = 3, unpaired two-tailed Student’s t-test). (D) Rescue of SidG toxicity by human Rac1. The rho5∆::TRP1 strain expressing Flag-SidG alone or co-expressing HA-Rac1 was spotted onto glucose or galactose plates. Lower panels: Immunoblots for indicated proteins. (TIF) [file ppat.1013957.s007.tif]

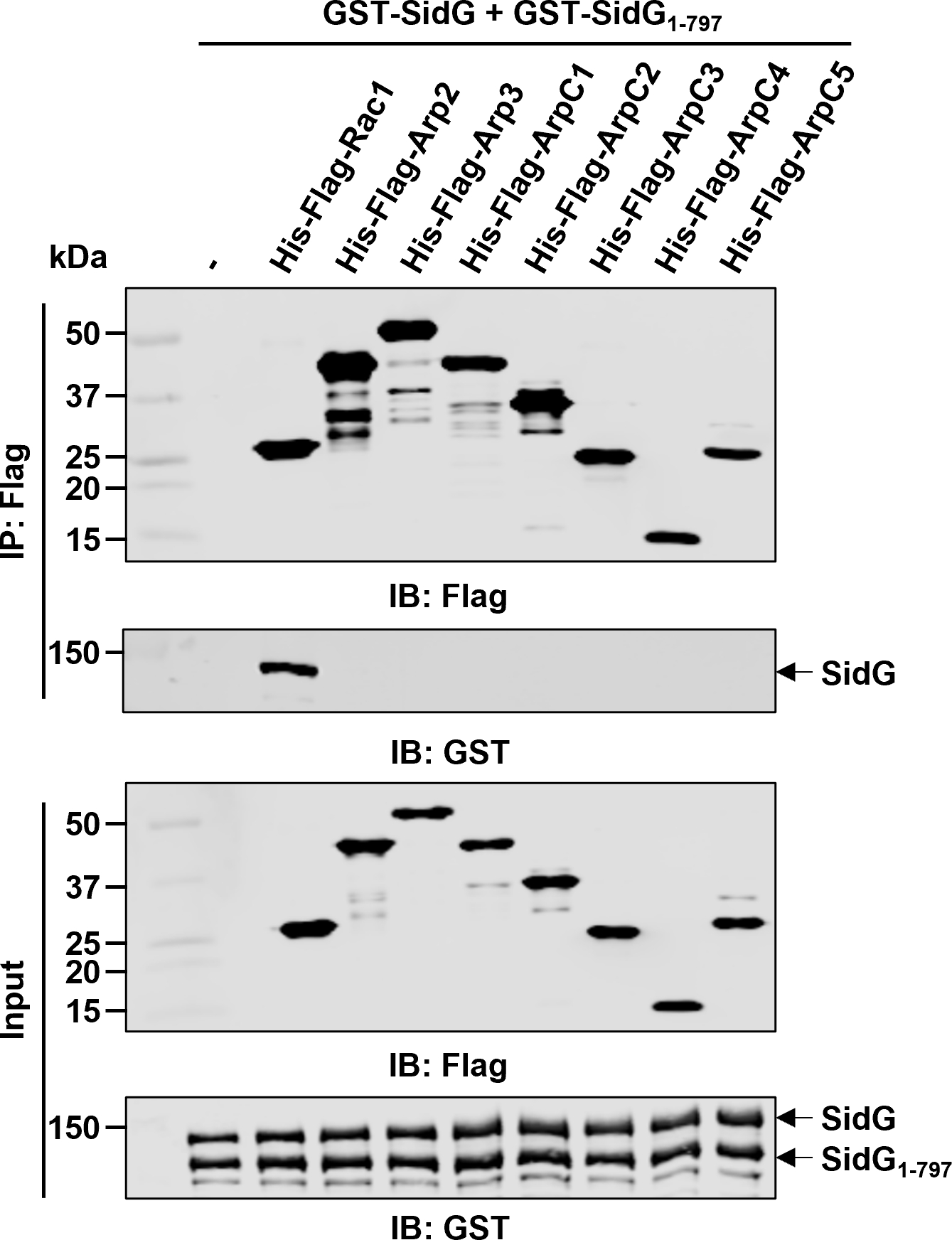

Supplement: S8 Fig — Lysate-based co-immunoprecipitation analysis of SidG binding to individual Arp2/3 subunits. Bacterial lysates containing a bait mixture of GST-SidG and GST-SidG1-797 were incubated with lysates containing His-Flag-tagged Rac1 or individual Arp2/3 subunits. Immunoprecipitated proteins by anti-Flag beads were analyzed by immunoblotting with anti-GST and anti-Flag antibodies. Data shown are representative of three independent experiments. (TIF) [file ppat.1013957.s008.tif]

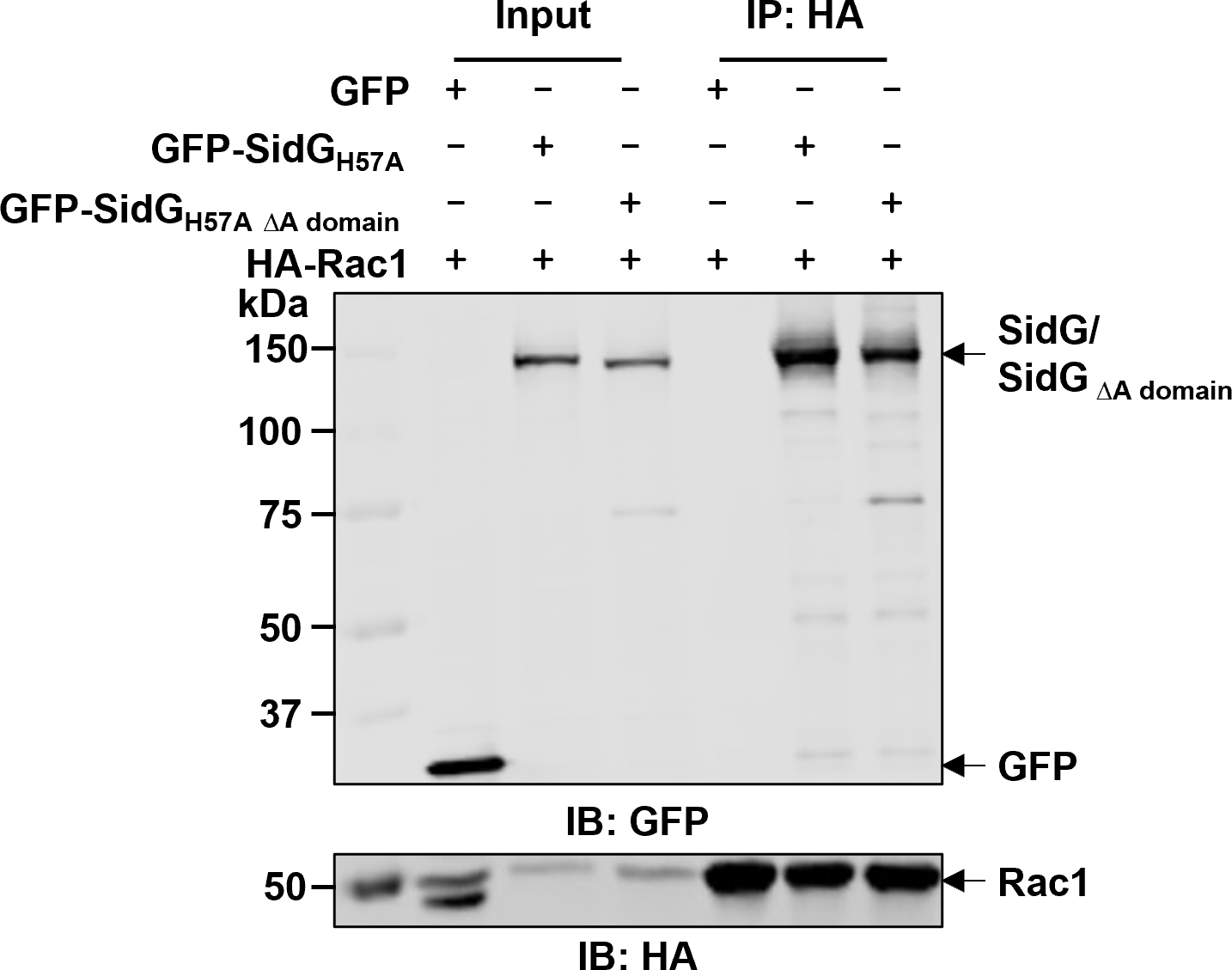

Supplement: S9 Fig — Co-immunoprecipitation of HA-Rac1 with GFP-SidGH57A or GFP-SidGH57A ΔA domain from co-transfected HEK293T cells. Immunoprecipitation was performed with anti-HA antibodies followed by immunoblotting with anti-HA and anti-GFP antibodies. Data shown are representative of three independent experiments. (TIF) [file ppat.1013957.s009.tif]

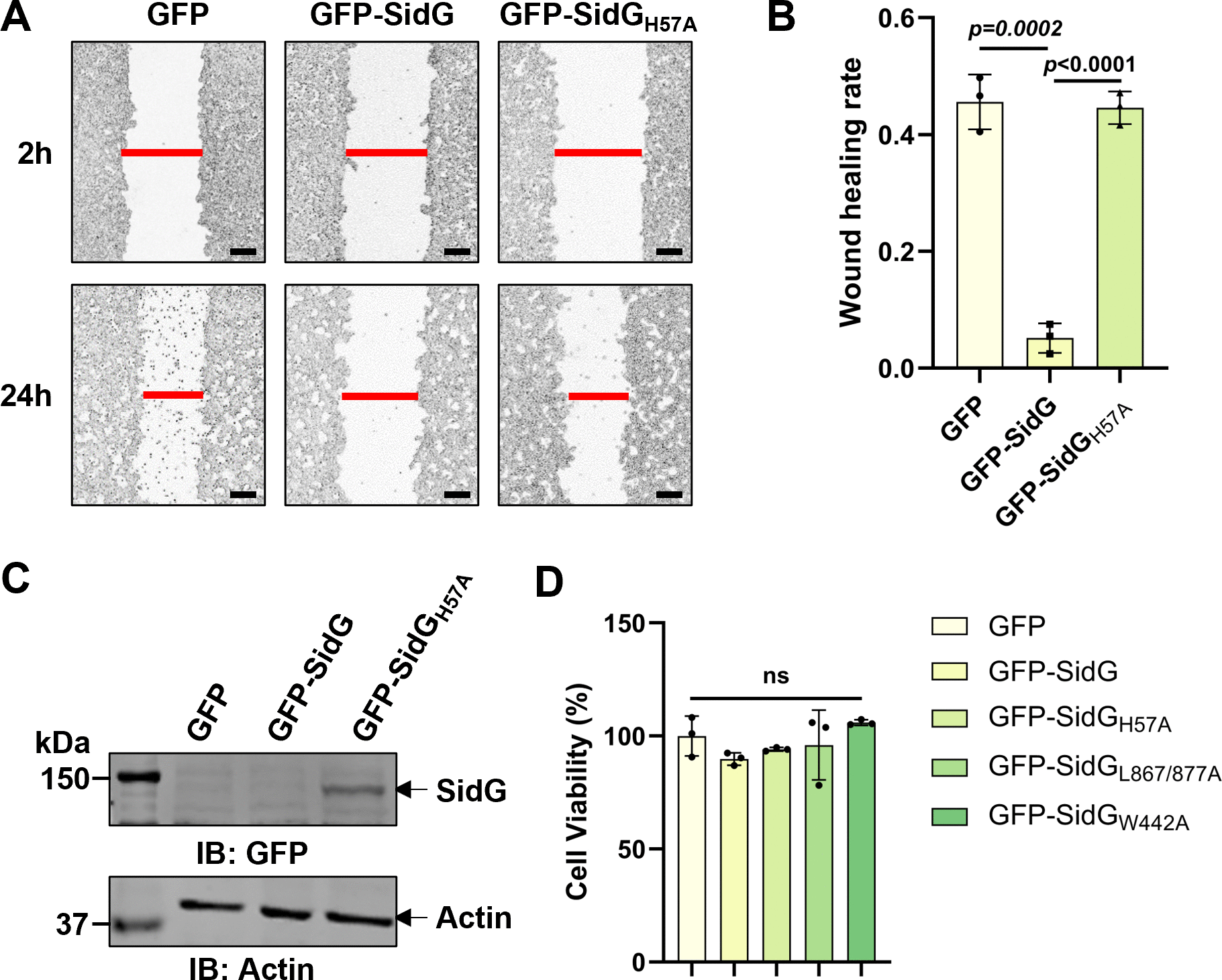

Supplement: S10 Fig — (A) HEK293 cells were scratched 24 h after transfection with GFP, GFP-SidG, or GFP-SidGH57A. Images were taken at 2 and 24 h post-scratch. Scale bar, 100 μm. (B) Quantification of the wound healing rate. Data are mean ± SD (n = 3) and analyzed by the unpaired two-tailed Student’s t-test. (C) Expression of GFP-SidG was detected by immunoblotting with anti-GFP antibodies. Actin served as a loading control. (D) Cell viability of HEK293T cells expressing the indicated proteins was assessed by CCK-8 assay at 24 h post-transfection. Data are mean ± SD (n = 3). ns, not significant. Data shown are representative of three independent experiments. (TIF) [file ppat.1013957.s010.tif]

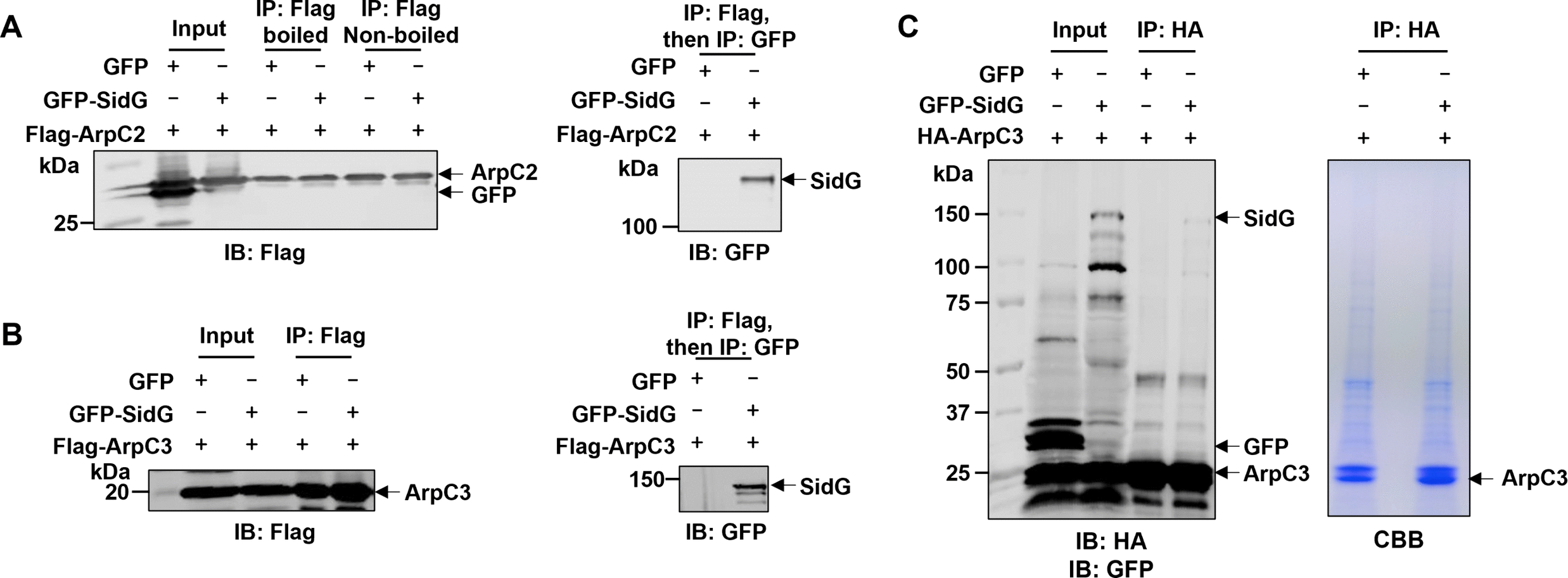

Supplement: S11 Fig — (A, B) Lysates from cells transfected with GFP-SidG were mixed with lysates prepared from cells expressing Flag-ArpC2 (A) or Flag-ArpC3 (B) and incubated at 30°C for 6 h. Samples were resolved by SDS-PAGE under non-boiled or boiled conditions and analyzed by immunoblotting. Right panels showed detection of GFP-SidG expression after sequential immunoprecipitation (anti-Flag, then anti-GFP). (C) Yeast cells co-expressing HA-ArpC3 and either GFP or GFP-SidG were induced with galactose overnight. Lysates were subjected to anti-HA immunoprecipitation followed by immunoblotting with anti-HA and anti-GFP antibodies (left) or Coomassie Brilliant Blue staining (right). Data shown are representative of three independent experiments. (TIF) [file ppat.1013957.s011.tif]

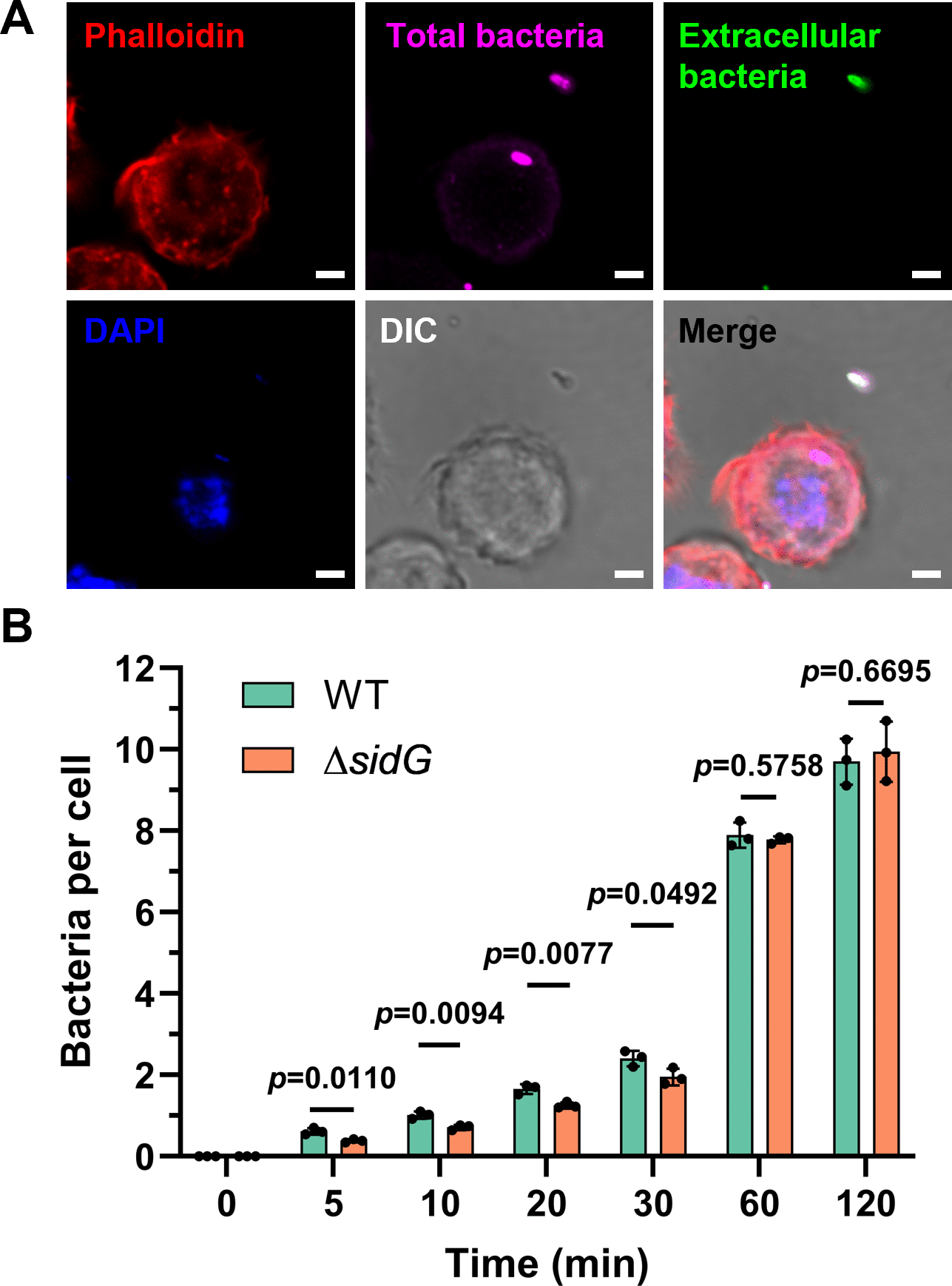

Supplement: S12 Fig — (A) Representative images of differential staining distinguishing extracellular (green) and total (magenta) bacteria. Scale bar, 2 μm. (B) Quantification of intracellular bacteria per cell over time in RAW 264.7 cells infected with WT or ∆sidG strains (MOI = 50). Data are mean ± SD (n = 3, at least 50 cells quantified per replicate) and analyzed by the unpaired two-tailed Student’s t-test. (TIF) [file ppat.1013957.s012.tif]

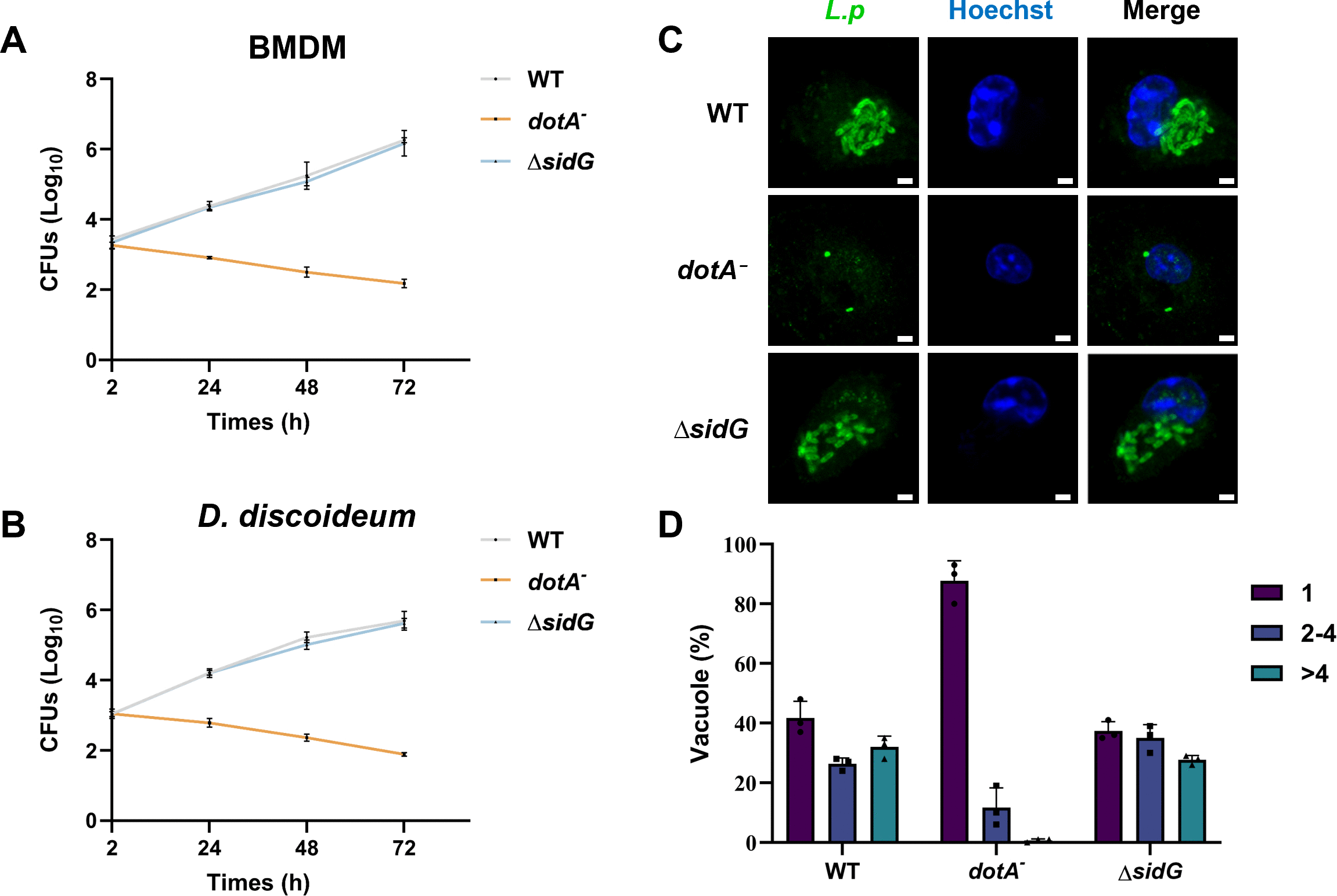

Supplement: S13 Fig — (A, B) Intracellular growth curves of the indicated L. pneumophila strains were performed in BMDMs (A) and D. discoideum (B). (C) Representative immunofluorescence images of BMDMs infected with the indicated L. pneumophila strains for 12 h at an MOI of 10. Bacteria were stained with the anti-L. pneumophila antibody (green). Nuclei were stained with Hoechst (blue). Scale bar, 2 μm. (D) Quantification of replicative vacuoles from the experiment in (C) at 12 h post-infection. Data are mean ± SD (n = 3, > 100 LCVs per condition per experiment). Uncropped scans of Western blots. (Uncropped scans of Western blots.pdf). (TIF) [file ppat.1013957.s013.tif]
