# Supplementary material for: Legionella effector protein SidG disrupts host cytoskeleton via targeting Arp2/3 complex
Source: PLoS Pathog. 2026 Feb 9;22(2):e1013957. doi: 10.1371/journal.ppat.1013957 (PMC12904589; doi:10.1371/journal.ppat.1013957)
Supplement: S1 Table — (DOCX) [file ppat.1013957.s014.docx]

**S1 Table. Bacterial and yeast strains used in this study.**

| Strains | Genotype, relevant markers | Reference |
| --- | --- | --- |
| *E. coli* |  | Our collection |
| BL21 Rosetta(DE3) | *F-ompT hsdSB*(*rB-mB-*) *gal dcm* (DE3) pRARE (*CamR*) | Our collection |
| DH5α(λpir) | supE44 d*lacU169*(φ80*lacZ∆M15*) *hsdR17 recA1 endA1 gyrA96 thi-1 relA1 pir tet*::*Mu recA* | Our collection |
| *L. pneumophila* |  |  |
| Lp02 | Philadelphia-1 *rpsL hsdR thyA* | Berger & Isberg, 1993 [1] |
| Lp03 | Lp02(*dotA*^-^) | Berger & Isberg, 1993 [1] |
| Lp02(pJB908) | Lp02(pJB908) | Liu & Luo, 2007 [2] |
| Lp03(pJB908) | Lp03(pJB908) | Liu & Luo, 2007 [2] |
| JY1001 | Lp02*∆sidG* | This study |
| JY1002 | Lp02*∆sidG*(pZL507) | This study |
| JY1003 | Lp02*∆sidG*(pZL507::*sidG*) | This study |
| JY1004 | Lp02*∆sidG*(pZL507::*sidG*_H57A_) | This study |
| JY1005 | Lp02*∆sidG*(pZL507::*sidG*_LH67A/L877A_) | This study |
| Yeast |  |  |
| W303 | *MATa/MATα {leu2-3,112 trp1-1 can1-100 ura3-1 ade2-1 his3-11,15} [phi+]* | Fan & Klein, 1996 [3] |
| W303 | *MATa/MATα {leu2-3,112 trp1-1 can1-100 ura3-1 ade2-1 his3-11,15} [phi+] rho5*∆::*TRP1* | This study |

**References**

[1] Berger KH, Isberg RR. Two distinct defects in intracellular growth complemented by a single genetic locus in *Legionella pneumophila*. Mol Microbiol. 1993;7(1):7-19. <https://doi.org/10.1111/j.1365-2958.1993.tb01092.x>. PMID: 8382332.

[2] Liu Y, Luo ZQ. The *Legionella pneumophila* effector SidJ is required for efficient recruitment of endoplasmic reticulum proteins to the bacterial phagosome. Infect Immun. 2007;75(2):592-603. <https://doi.org/10.1128/iai.01278-06>. PMID: 17101649.

[3] Fan HY, Cheng KK, Klein HL. Mutations in the RNA polymerase II transcription machinery suppress the hyperrecombination mutant hpr1 delta of *Saccharomyces cerevisiae*. Genetics. 1996;142(3):749-59. <https://doi.org/10.1093/genetics/142.3.749>. PMID: 8849885.
