# Supplementary material for: Legionella effector protein SidG disrupts host cytoskeleton via targeting Arp2/3 complex
Source: PLoS Pathog. 2026 Feb 9;22(2):e1013957. doi: 10.1371/journal.ppat.1013957 (PMC12904589; doi:10.1371/journal.ppat.1013957)
Supplement: S2 Table — (DOCX) [file ppat.1013957.s015.docx]

**S2 Table. Plasmids used in this study.**

| Plasmid | Relevant phenotypes | Sources |
| --- | --- | --- |
| pSR47s | *ori*R6K, *ori*T RP4, Kan^R^, *SacB* | Dumenil *et al.*, 2001 [1] |
| pJB908 | Amp, *thy*^+^ | Bardill *et al.*, 2005 [2] |
| pZL507 | For expression His_6_-tagged protein *L. pneumophila* | Xu *et al.*, 2010 [3] |
| pEGFPC-1 | For expressing C-terminal GFP fusion proteins | Clontech |
| pCMV-Flag | For expressing C-terminal Flag fusion proteins | Li *et al.*, 2021 [4] |
| pCMV-HA | For expressing C-terminal HA fusion proteins | Our collection |
| pGEX-6P-1 | For purification GST-tagged protein | Our collection |
| pSB157m | Amp, *ura^+^,* GAL promoter | Tan *et al.*, 2011 [5] |
| p425GPD | Amp, *leu*^+^, GAD promoter | Mumberg *et al.*, 1995 [6] |
| pJYΔ*sidG* | Construct used for in-frame deletion of *sidG* | This study |
| pJY1101 | pSB157m-*sidG* | This study |
| pJY1102 | pSB157m-*sidG*_H57A_ | This study |
| pJY1103 | pSB157m-*sidG*_C623A_ | This study |
| pJY1104 | pSB157m-*sidG*_W32A_ | This study |
| pJY1105 | pSB157m-*sidG*_W148A_ | This study |
| pJY1106 | pSB157m-*sidG*_D157A_ | This study |
| pJY1107 | pSB157m-*sidG*_D158A_ | This study |
| pJY1108 | pSB157m-*sidG_LS_* | This study |
| pJY1109 | pSB157m-*sidG_LB_* | This study |
| pJY1110 | pSB157m-*sidG_LS_* _H48A_ | This study |
| pJY1111 | pSB157m-*sidG_LS_* _D148A_ | This study |
| pJY1112 | pSB157m-*sidG_LS_* _C613A_ | This study |
| pJY1113 | pSB157m-*sidG*_L867A/L877A_ | This study |
| pJY1114 | pSB157m-*sidG*_∆A domain_ | This study |
| pJY1115 | p425GPD-*RAC1* | This study |
| pJY1116 | p425GPD-*RAC1*_Q61L_ | This study |
| pJY1117 | p425GPD-*RAC1*_T17N_ | This study |
| pJY1118 | p425GPD-*ARP2* | This study |
| pJY1119 | p425GPD-*ARP3* | This study |
| pJY1120 | p425GPD-*ARPC1* | This study |
| pJY1121 | p425GPD-*ARPC2* | This study |
| pJY1122 | p425GPD-*ARPC3* | This study |
| pJY1123 | p425GPD-*ARPC4* | This study |
| pJY1124 | p425GPD-*ARPC5* | This study |
| pJY1125 | pCMV-Flag-*sidG*_H57A_ | This study |
| pJY1126 | pCMV-Flag-*sidG*_H57A/L867A/L877A_ | This study |
| pJY1127 | pCMV-Flag-*RAC1* | This study |
| pJY1128 | pCMV-Flag-*ARP2* | This study |
| pJY1129 | pCMV-Flag-*ARP3* | This study |
| pJY1130 | pCMV-Flag-*ARPC2* | This study |
| pJY1131 | pCMV-Flag-*ARPC3* | This study |
| pJY1132 | pCMV-Flag-*WASF1* | This study |
| pJY1133 | pGEX-6P-1-*RAC1* | This study |
| pJY1134 | pGEX-6P-1-*PBD* | This study |
| pJY1135 | pCMV-HA-*RAC3* | This study |
| pJY1136 | pCMV-HA-*RAC1* | This study |
| pJY1137 | pCMV-HA-*RAC1*_Q61L_ | This study |
| pJY1138 | pCMV-HA-*RAC1*_T17N_ | This study |
| pJY1139 | pCMV-HA-*CDC42* | This study |
| pJY1140 | pCMV-HA-*RHOA* | This study |
| pJY1141 | pEGFPC-*sidG* | This study |
| pJY1142 | pEGFPC-*sidG*_H57A_ | This study |
| pJY1143 | pEGFPC-*sidG*_504-973_ | This study |
| pJY1144 | pEGFPC-*sidG*_700-973_ | This study |
| pJY1145 | pEGFPC-*sidG*_504-750_ | This study |
| pJY1146 | pEGFPC-*sidG*_1-510_ | This study |
| pJY1147 | pEGFPC-*sidG*_1-797_ | This study |
| pJY1148 | pEGFPC-*sidG*_H57A/L867A_ | This study |
| pJY1149 | pEGFPC-*sidG*_H57A/L877A_ | This study |
| pJY1150 | pEGFPC-*sidG*_H57A/V927A_ | This study |
| pJY1151 | pEGFPC-*sidG*_H57A/E821A_ | This study |
| pJY1152 | pEGFPC-*sidG*_H57A/D883A_ | This study |
| pJY1153 | pEGFPC-*sidG*_H57A/E821A/D836A_ | This study |
| pJY1154 | pEGFPC-*sidG*_H57A/D883A/D836A_ | This study |
| pJY1155 | pEGFPC-*sidG*_H57A/E821A/D836A/D883A_ | This study |
| pJY1156 | pEGFPC-*sidG*_1-797 H57A_ | This study |
| pJY1157 | pEGFPC-*sidG*_H57A ΔA domain_ | This study |
| pJY1158 | pEGFPC-*sidG*_A domain_ | This study |
| pJY1159 | pEGFPC-*sidG*_L867A/L867A_ | This study |
| pJY1160 | pEGFPC-*sidG*_W442A_ | This study |
| pJY1161 | pZL507-*sidG* | This study |
| pJY1162 | pZL507-*sidG*_H57A_ | This study |
| pJY1163 | pZL507-*sidG*_L867A/L877A_ | This study |

**References**

[1] Duménil G, Isberg RR. The *Legionella pneumophila* IcmR protein exhibits chaperone activity for IcmQ by preventing its participation in high-molecular-weight complexes. Mol Microbiol. 2001;40(5):1113-27. <https://doi.org/10.1046/j.1365-2958.2001.02454.x>. PMID: 11401716.

[2] Bardill JP, Miller JL, Vogel JP. IcmS-dependent translocation of SdeA into macrophages by the *Legionella pneumophila* type IV secretion system. Mol Microbiol. 2005;56(1):90-103. <https://doi.org/10.1111/j.1365-2958.2005.04539.x>. PMID: 15773981.

[3] Xu L, Shen X, Bryan A, Banga S, Swanson MS, Luo ZQ. Inhibition of host vacuolar H^+^-ATPase activity by a *Legionella pneumophila* effector. PLoS Pathog. 2010;6(3):e1000822. <https://doi.org/10.1371/journal.ppat.1000822>. PMID: 20333253.

[4] Li G, Liu H, Luo ZQ, Qiu J. Modulation of phagosome phosphoinositide dynamics by a *Legionella* phosphoinositide 3-kinase. EMBO Rep. 2021;22(3):e51163. <https://doi.org/10.15252/embr.202051163>. PMID: 33492731.

[5] Tan Y, Luo ZQ. *Legionella pneumophila* SidD is a deAMPylase that modifies Rab1. Nature. 2011;475(7357):506-9. <https://doi.org/10.1038/nature10307>. PMID: 21734656.

[6] Mumberg D, Müller R, Funk M. Yeast vectors for the controlled expression of heterologous proteins in different genetic backgrounds. Gene. 1995;156(1):119-22. <https://doi.org/10.1016/0378-1119(95)00037-7>. PMID: 7737504.
