# Supplementary material for: Legionella effector protein SidG disrupts host cytoskeleton via targeting Arp2/3 complex
Source: PLoS Pathog. 2026 Feb 9;22(2):e1013957. doi: 10.1371/journal.ppat.1013957 (PMC12904589; doi:10.1371/journal.ppat.1013957)
Supplement: S3 Table — (DOCX) [file ppat.1013957.s016.docx]

**S3 Table. Primers used in this study.**

| Primer | Sequence | Note |
| --- | --- | --- |
| PY1201 | CGCAGATCTatgtttttacccaaag | *sidG* 5’BglII |
| PY1202 | CGCGTCGACttatatgcgcatcgtactt | *sidG* 3’SalI |
| PY1203 | tggtggggatgtaggtGCtgcttccattaatatga | *sidG*(H57A)-1 |
| PY1204 | tcatattaatggaagcaGCacctacatccccacca | *sidG*(H57A)-2 |
| PY1205 | aatttacataccaataacGCctcacttacttcgataga | *sidG*(C623A)-1 |
| PY1206 | tctatcgaagtaagtgagGCgttattggtatgtaaatt | *sidG*(C623A)-2 |
| PY1207 | ggtattacagtccaaacaGCgggaactaatgatcgaccg | *sidG*(W32A)-1 |
| PY1208 | cggtcgatcattagttcccGCtgtttggactgtaatacc | *sidG*(W32A)-2 |
| PY1209 | gacatagattggagttggGCgccgggaagattacagaat | *sidG*(W148A)-1 |
| PY1210 | attctgtaatcttcccggcGCccaactccaatctatgtc | *sidG*(W148A)-2 |
| PY1211 | agattacagaatacagaagCAgacatggtttgggaaaga | *sidG*(D157A)-1 |
| PY1212 | tctttcccaaaccatgtcTGcttctgtattctgtaatct | *sidG*(D157A)-2 |
| PY1213 | ttacagaatacagaagatgCAatggtttgggaaagagag | *sidG*(D158A)-1 |
| PY1214 | ctctctttcccaaaccatTGcatcttctgtattctgtaa | *sidG*(D158A)-2 |
| PY1215 | ctggaagaggctctaGCggaagaaggaattgat | *sidG*(W442A)-1 |
| PY1216 | atcaattccttcttccGCtagagcctcttccag | *sidG*(W442A)-2 |
| PY1217 | CGCAGATCTatgattgactctccatgc | *sidG*(504-973 aa) 5’BglII |
| PY1218 | CGCGTCGACttaaacttggcatggaga | *sidG*(1-510 aa) 3’SalI |
| PY1219 | CGCAGATCTatggatcctgaagcc | *sidG*(700-973 aa) 5’BglII |
| PY1220 | CGCGTCGACttaatagacgaggtttaa | *sidG*(504-750 aa) 3’SalI |
| PY1221 | CGCGGATCCgtaaaatcctgagatac | *sidG* upF BamHI knock-out |
| PY1222 | ttctatttgatgttcgagagaatcatttgc | *sidG* upR knock-out |
| PY1223 | gcaaatgattctctcgaacatcaaatagaa | *sidG* downF knock-out |
| PY1224 | CGCGTCGACaccaagttcattgaa | *sidG* downR sal1 knock-out |
| PY1225 | CGCAGATCTatggctgccatccgga | *RHOA* 5’BglII |
| PY1226 | CGCGTCGACtcacaagacaaggcac | *RHOA* 3’SalI |
| PY1227 | CGCAGATCTatgcagacaattaagt | *CDC42* 5’BglII |
| PY1228 | CGCGTCGACtcatagcagcacacac | *CDC42* 3’SalI |
| PY1229 | CGCAGATCTatgcaggccatcaagt | *RAC1* 5’BglII |
| PY1230 | CGCGTCGACttacaacagcaggcat | *RAC1* 3’SalI |
| PY1231 | CGCGGATCCatgcaggccatcaag | *RAC3* 5’BamHI |
| PY1232 | CGCGTCGACctagaagacggtgca | *RAC3* 3’SalI |
| PY1233 | CGCGGATCCatggacagccagggca | *ARP2* 5’BamHI |
| PY1234 | CGCGTCGACttatcgaacagtcacac | *ARP2* 3’SalI |
| PY1235 | CGCGGATCCatggcgggacggctgc | *ARP3* 5’BamHI |
| PY1236 | CGCGTCGACttacgacatgactccaa | *ARP3* 3’SalI |
| PY1237 | CGCGGATCCatgtcactgcatcagt | *ARPC1A* 5’BamHI |
| PY1238 | CGCGTCGACtcacattatccggagg | *ARPC1A* 3’SalI |
| PY1239 | CGCGGATCCatgatcctgctggagg | *ARPC2* 5’BamHI |
| PY1240 | CGCGTCGACttagcgggatgaaaac | *ARPC2* 3’SalI |
| PY1241 | CGCAGATCTatgccggcttaccact | *ARPC3* 5’BglII |
| PY1242 | CGCGTCGACtcactgtccaggtcct | *ARPC3* 3’SalI |
| PY1243 | CGCGGATCCatgcgcttcatgatga | *ARPC4* 5’BamHI |
| PY1244 | CGCGTCGACttaaaaattcttaaggaactct | *ARPC4* 3’SalI |
| PY1245 | CGCGGATCCatgtcgaagaacacag | *ARPC5* 5’BamHI |
| PY1246 | CGCGTCGACctacacagtttttctt | *ARPC5* 3’SalI |
| PY1247 | CCAGATAATGGAAAGTAGCA | *RHO5* upF knock-out |
| PY1248 | atatatatagtaatgtcgttCAGACTTATTAGTATA CT | *RHO5* upR knock-out |
| PY1249 | AGTATACTAATAAGTCTGaacgacattactata tatat | *TRP1*F |
| PY1250 | ACTTTTTCTGCCTCCTTGATAcctgatgcggta ttttctcc | *TRP1*R |
| PY1251 | ggagaaaataccgcatcaggTATCAAGGAGGCA GAGAAAAAGT | RHO5 downF knock-out |
| PY1252 | AGATCATCTAAGGATGGGCTGG | *RHO5* downR knock-out |
| PY1253 | TTGAACAGGCGGTGAGGTAC | *RHO5*F qPCR |
| PY1254 | GGTGTTGGTTGTTGCAGTCG | *RHO5*R qPCR |
| PY1255 | GAGAGCCCCGAAAGCTTACA | *TRP1*F qPCR |
| PY1256 | GTCTCCACACCTCCGCTTAC | *TRP1*R qPCR |

Restriction sites were underlined.
