# Supplementary material for: Legionella effector protein SidG disrupts host cytoskeleton via targeting Arp2/3 complex
Source: PLoS Pathog. 2026 Feb 9;22(2):e1013957. doi: 10.1371/journal.ppat.1013957 (PMC12904589; doi:10.1371/journal.ppat.1013957)
Supplement: S4 Table — (DOCX) [file ppat.1013957.s017.docx]

**S4 Table. Antibodies used in this study.**

| Antibodies | Source | Identifier |
| --- | --- | --- |
| Mouse anti-Flag | ABclonal | Cat# AE005 |
| Mouse anti-HA | ABclonal | Cat# AE008 |
| Mouse anti-GFP | ABclonal | Cat# AE012 |
| Rabbit anti-Arp3 | Proteintech | Cat# 13822-1-AP |
| Rabbit anti-Rac | Abcam | Cat# ab97732 |
| Rabbit anti-PGK1 | Abcam | Cat# EPR19057 |
| Rabbit anti-GST | Proteintech | Cat# 10000-0-AP |
| Rabbit anti-Actin | Servicebio | Cat# GB15003-100 |
| Rabbit anti-isocitrate dehydrogenase (ICDH) | Sigma-Aldrich | Cat# ABS2090 |
| Rabbit anti-Legionella | Qiu *et al.*, 2016[1] | N/A |
| Goat Anti-Rabbit IgG H&L  (Alexa Fluor® 790) | Abcam | Cat# ab175781 |
| Goat Anti-Mouse IgG H&L (Alexa Fluor® 680) | Abcam | Cat# ab175775 |

**References**

1. Qiu J, Sheedlo MJ, Yu K, Tan Y, Nakayasu ES, Das C, et al. Ubiquitination independent of E1 and E2 enzymes by bacterial effectors. Nature. 2016;533(7601):120-4. <https://doi.org/10.1038/nature17657>. PMID: 27049943.
